# Presynaptic Kainate Receptors onto Somatostatin Interneurons Are Recruited by Activity throughout Development and Contribute to Cortical Sensory Adaptation

**DOI:** 10.1523/JNEUROSCI.1461-22.2023

**Published:** 2023-10-25

**Authors:** Tevye J. Stachniak, Ali Ö. Argunsah, Jenq-Wei Yang, Linbi Cai, Theofanis Karayannis

**Affiliations:** Laboratory of Neural Circuit Assembly, Brain Research Institute, Neuroscience Center Zurich, University of Zurich, CH-8057 Zurich, Switzerland

**Keywords:** adaptation, barrel cortex, inhibition, kainate, somatosensory, synapse

## Abstract

Somatostatin (SST) interneurons produce delayed inhibition because of the short-term facilitation of their excitatory inputs created by the expression of metabotropic glutamate receptor 7 (mGluR7) and presynaptic GluK2-containing kainate receptors (GluK2-KARs). Using mice of both sexes, we find that as synaptic facilitation at layer (L)2/3 SST cell inputs increases during the first few postnatal weeks, so does GluK2-KAR expression. Removal of sensory input by whisker trimming does not affect mGluR7 but prevents the emergence of presynaptic GluK2-KARs, which can be restored by allowing whisker regrowth or by acute calmodulin activation. Conversely, late trimming or acute inhibition of Ca^2+^/calmodulin-dependent protein kinase II is sufficient to reduce GluK2-KAR activity. This developmental and activity-dependent regulation also produces a specific reduction of L4 GluK2-KARs that advances in parallel with the maturation of sensory processing in L2/3. Finally, we find that removal of both GluK2-KARs and mGluR7 from the synapse eliminates short-term facilitation and reduces sensory adaptation to repetitive stimuli, first in L4 of somatosensory cortex, then later in development in L2/3. The dynamic regulation of presynaptic GluK2-KARs potentially allows for flexible scaling of late inhibition and sensory adaptation.

**SIGNIFICANCE STATEMENT** Excitatory synapses onto somatostatin (SST) interneurons express presynaptic, calcium-permeable kainate receptors containing the GluK2 subunit (GluK2-KARs), activated by high-frequency activity. In this study we find that their presence on L2/3 SST synapses in the barrel cortex is not based on a hardwired genetic program but instead is regulated by sensory activity, in contrast to that of mGluR7. Thus, in addition to standard synaptic potentiation and depression mechanisms, excitatory synapses onto SST neurons undergo an activity-dependent presynaptic modulation that uses GluK2-KARs. Further, we present evidence that loss of the frequency-dependent synaptic components (both GluK2-KARs and mGluR7 via Elfn1 deletion) contributes to a decrease in the sensory adaptation commonly seen on repetitive stimulus presentation.

## Introduction

The development of interneuron diversity in the cortex expands the computational capacity of cortical circuits by using an array of cell-type-specific electrophysiological and morphologic properties. An archetypical example is the cell-specific expression of synaptic proteins onto somatostatin (SST)-positive interneurons that tune these inhibitory cells toward high-frequency delayed recruitment. This delayed recruitment of SST cells leads to late cortical inhibition, which is very distinct from the rapid feedforward inhibition characteristic of parvalbumin (PV)-positive interneurons (Pouille and Scanziani, 2004; Silberberg and Markram, 2007). A key molecular determinant of the delayed recruitment of SST cells is the expression of the protein Extracellular Leucine Rich Repeat and Fibronectin type III Domain Containing 1 (Elfn1).

*In vitro* electrophysiological studies have shown that the loss of the facilitating properties of pyramidal (Pyr)→SST synapses on removal of Elfn1 results in a rapid recruitment of SST cells (Sylwestrak and Ghosh, 2012). The precise timing of SST inhibition is sacrificed for an earlier inhibitory wave, potentially affecting sensory stimulus processing *in vivo*. Postsynaptic Elfn1 acts via the presynaptic recruitment of constitutively activated metabotropic glutamate receptor 7 (mGluR7) and late-activated kainate receptors (KARs; Sylwestrak and Ghosh, 2012; Tomioka et al., 2014; Stachniak et al., 2019). The addition of presynaptic kainate receptors containing the GluK2 subunit (GluK2-KARs) strengthens SST interneuron recruitment by contributing to a late increase in neurotransmitter release (Sylwestrak and Ghosh, 2012). Similarly, mossy fiber synapses engage presynaptic GluK2-KARs to amplify synaptic transmission by increasing presynaptic calcium levels (Contractor et al., 2001; Dietmar et al., 2001; Pinheiro et al., 2007; Sachidhanandam et al., 2009). Expression of GluK2-KARs at Pyr→SST synapses reduces the number of stimuli required to recruit SST interneurons without impairing the frequency-dependent properties of late inhibition (Stachniak et al., 2019). Conversely, induction of long-term plasticity (LTP) at CA3→CA1 synapses reduces presynaptic KAR-dependent synaptic facilitation (Sallert et al., 2009). Regulation of presynaptic GluK2-KAR activity therefore represents a means to modulate SST neuron recruitment without completely impairing facilitation, thus to some extent sparing the synaptic timing and the late inhibitory properties of SST interneurons.

Although the purpose of late inhibition is not well understood, evidence suggests that it serves to mitigate excitability of sensory responses to repetitive stimuli, thereby creating sensory adaptation (Natan et al., 2017; Seay et al., 2020). It was reported that SST, but not PV, interneurons provide inhibition of responses to adapted stimuli (Natan et al., 2017). Interestingly, it has been shown that activity in the L1 dendritic tufts of pyramidal cells in sensory regions is linked to perception, and consequently SST-mediated inhibition regulating the activity in distal dendrites can modulate the perception of sensory inputs (Takahashi et al., 2016; Schulz et al., 2018). Therefore, the frequency-dependent delayed inhibition produced by SST neurons may play a role in limiting recurrent activity and extraneous sensory information during repeatedly presented sensory stimuli. Thus, the developmental emergence of refined cortical representations of the sensory environment may require the regulation of the late-phase cortical activity provided by SST cells.

Indeed, sensory adaptation develops over the first 2 postnatal weeks, a period in which major changes in sensory-evoked activity take place as mice switch the usage of their whiskers from passive interactions to active whisking (Borgdorff et al., 2007; Leighton and Lohmann, 2016; van der Bourg et al., 2019). It has been reported that during the first weeks of postnatal life, mice display an initial increase in multiunit activity (MUA) in barrel cortex in response to a single-whisker touch, which includes a substantial amount of late-phase spiking (50–1000 ms) at around postnatal day (P)7 (van der Bourg et al., 2019; Cai et al., 2022). This late-phase sensory-evoked activity declines in juvenile mice, becoming more compressed as cortex matures to the adult state. Interestingly, although repetitive deflections of a single whisker in adult mice lead to attenuation of barrel cortex activation, during early development they lead to facilitation, possibly because of the expansion of recurrent local excitation and the concomitant lack of inhibition (Borgdorff et al., 2007).

In this study, we reveal a developmental increase in presynaptic recruitment of GluK2-KAR in excitatory synapses onto layer (L)2/3 SST cells of the barrel cortex during the second postnatal week. We further find that the expression of presynaptic KARs is layer dependent and sensory activity dependent and is mediated by Ca^2+^/calmodulin-dependent protein kinase II (CaMKII) activation. Finally, we reveal that the dynamic regulation of KARs supports developmental changes in SST cell recruitment, which contributes to the adaptation of barrel cortex spiking to repetitive whisker stimuli *in vivo*.

## Materials and Methods

### Mice

All animal experiments were conducted according to Swiss Laboratory Animal Science Association guidelines for animal research and approved by the Cantonal Veterinary Office Zurich. Animals were housed on an inverted 12 h light/dark (21:00/09:00) cycle with *ad libitum* access to water and mouse chow. Postnatal age is recorded with date of birth as P0. Animal lines used in this study are *SSTCre* (Sst^<tm2.1(cre)Zjh/J^), *Ai14* (B6;129S6-Gt(ROSA)^26Sortm14(CAG-tdTomato)Hze/J^), and *Elfn1KO* (Elfn1^tm1(KOMP)Vlcg^). Both male and female mice were used in cortical brain slice physiology and *in vivo* physiology experiments.

### *In vitro* electrophysiology

Whole-cell patch-clamp electrophysiological recordings were performed on fluorescent-labeled SST neurons located in neocortical L2/3 and L4 of barrel cortex (approximately, bregma −0.5 to −2.0 mm) in acute brain slices prepared from postnatal day 7–22 (P7–P22) male and female mice. Coronal brain slices from barrel cortex were prepared in cold artificial CSF (ACSF) containing the following (in mm): 128 NaCl, 26 NaHCO3, 10 D-glucose, 3 KCl, 1 MgCl2, 2 CaCl2, and 1.25 NaH_2_PO_4_, aerated with 95% O_2_/5% CO_2_. Acute slices were perfused at a rate of 2–3 ml/min with oxygenated ACSF at room temperature. Patch electrodes were made from borosilicate glass (Harvard Apparatus) and had a resistance of 2–4 MΩ. The intracellular solution contained the following (in mm): 125 K gluconate, 2 KCl, 10 HEPES, 10 phosphocreatine, 4 MgCl2, 1 EGTA, 0.1 CaCl2, 4 ATP, and 0.4 GTP, pH 7.35, 290 mOsm.

Experiments were performed in voltage-clamp mode using the AxoPatch 200B amplifier (Molecular Devices). Visually guided patch clamp of fluorescent-labeled cells was performed on a Zeiss Axioscope using a Retiga Electro camera (01-ELECTRO-M-14-C-OC, Teledyne Scientific & Imaging). Access resistance was monitored to ensure the stability of recording conditions. Recordings with access resistance >40 MΩ or whole-cell capacitance <4 pF were excluded. No compensation was made for access resistance, and no correction was made for the junction potential between the pipette and the ACSF. Following a baseline stabilization period (2–3 min), evoked synaptic currents recorded in 2 min (12 sweeps) at V_h_ = −70 mV were averaged and analyzed using Clampfit 10 software (Molecular Devices). Five electrical stimuli from a Digitimer isolated stimulator (DS2A Mk.II) were delivered at 50 Hz through a monopolar glass pipette (2–4 MΩ) positioned in L2/3, close to the soma of the recorded cells. The stimulating electrode was placed typically 100-250 μm from the recorded cell, parallel to the pial surface. A similar distance was maintained in L4 recordings, with the stimulating electrode parallel to the L2/3–L4 boundary. For the L2/3 to L4 recordings, the simulating electrode was then moved to lower L3 within the same column, again ∼100–250 μm from the recorded cell but offset slightly to avoid directly stimulating the axon of the recorded cell. Stimulation intensity and duration were adjusted to produce stable evoked excitatory postsynaptic current (EPSC) amplitudes.

Bath-applied compounds NS102 (20 mm; catalog #N179, Sigma-Aldrich), UBP310 (10 mm; catalog #3621, Tocris Bioscience) and KN-62 (10 mm, catalog #1277, Tocris Bioscience) were dissolved as 1:1000 stock solutions in DMSO; MSOP (50 mm; catalog #0803/5, Tocris Bioscience) and CALP3 (100 mm; catalog #V5640, Sigma-Aldrich), were dissolved in water. Recirculating KN-62 was applied for at least 15 min to allow Pyr→SST synapses to stabilize before NS102 testing. CALP3 was applied for 3 min, then slices were washed with ACSF for at least 25 min to allow synapses to reach steady state before NS102 application.

### Animal surgery

We used *Elfn1KO* and WT littermates at the age of P11–P30 for the multielectrode recordings. Mice were anesthetized by urethane (1.5 g/1 kg) throughout the whole experiment. A heating pad was used to maintain the mouse body temperature at 37°C. The depth of anesthesia was checked with breathing speed and paw reflexes throughout the experiment.

The skull of the right hemisphere was exposed by removing the skin on top, and a metallic head holder was implanted on the skull with cyanoacrylate glue and dental cement. A 20 gauge needle was used to open an ∼3 mm × 3 mm cranial window that exposed the S1 (wS1) barrel field. Extreme care was taken not to cause damage or surface bleeding during surgery.

### Whisker stimulation

A single whisker was stimulated 1 mm from the snout in a rostral-to-caudal direction (∼1 mm displacement) using a stainless steel rod (1 mm diameter) connected to a miniature solenoid actuator. The movement of the tip of the stimulator bar was measured precisely using a laser micrometer (MX SERIES, Metralight) with a 2500 Hz sampling rate. The stimulus takes 26 ms to reach the maximal 1 mm whisker displacement, with a total duration of 60 ms until it reaches baseline (Yang et al., 2017). The whisker was stimulated at 10 Hz for 2 s.

### *In vivo* multielectrode recordings

The wS1 neural activities were recorded with an eight-shank 64-channel silicon probe. Each of the eight shanks has eight recording sites (100 µm apart). The distance between each shank is 200 µm (NeuroNexus Technologies). The silicon probe was labeled with 1,1′-dioctadecyl-3,3,3′,3′-tetramethylindocarbocyanine (DiI; Invitrogen) and inserted perpendicularly into the barrel cortex. A silver wire was placed into the cerebellum as a ground electrode. All data were acquired at 20 kHz and stored with MC_RACK software (Multichannel Systems). The total duration of multielectrode recordings varied between 3 and 5 h. After each experiment, the animal was deeply anesthetized by ketamine (120 mg/kg, ketamine, 50 mg/ml; hameln pharma) and perfused through the aorta with ringer solution. The brain was kept in 4% PFA. Tangential sections (200 μm thick) were prepared for cytochrome-oxidase (CO) histochemistry. By combining the DiI and CO staining, the insertion position of the eight shank probes were identified in the barrel cortex. Only the shanks located within the identified individual barrels were used for the data analysis.

### Experimental design and statistical analysis

#### Analysis of *in vivo* multielectrode silicon probe data

Extracellular silicon probe data were analyzed using a custom-made MATLAB script (version 2019a, MathWorks). The raw data signal was bandpass filtered (0.8–5 kHz), and the MUA was extracted with the threshold of 7.5 times the SD of baseline.

The current source density (CSD) map was used to identify L2/3 and L4. The earliest CSD sink was identified as layer 4, followed by L2/3 (Reyes-Puerta et al., 2015; van der Bourg et al., 2017). The MUA was smoothed using a Gaussian kernel (0 mean, 5 ms sigma) and averaged across barrels over 20 trials for each stimulation pulse separately. Each pulse contains an ∼90-ms-long epoch following whisker stimulation pulse onset. Mean firing rate was calculated in two time windows after each whisker deflection. For analysis of the early window (the first 50 ms following each whisker stimulus at 10 Hz) and the late window (the last 50 ms following each whisker stimulus at 10 Hz), average spikes rates across the 2 s response train (average of responses 3–20) were normalized to the initial response, averaged from the first two stimuli (average of responses 1–2). Average baseline was calculated from the 200 ms window before stimulus onset. Adaptation was calculated as [average (early or late) activity – baseline] / [average (early or late) initial activity], such that a return to baseline after the initial response would constitute 100% adaptation. Number of measurements *n*/*N* indicates *n* channels recorded from *N* animals, using 1 channel per barrel for each whisker stimulated. Comparing early and late adaptation within layers, a two-way ANOVA was followed with a Bonferroni *post hoc* test. Statistical outcomes are represented in figures as follows: n.s., *p* > 0.05, *p < 0.05, **p < 0.01, and ***p < 0.001.

#### Electrophysiology data analysis

Values are represented as mean ± SEM. Statistical comparisons use cells or electrodes, as (*n*) number of measurements, which are reported as *n*/*N*, to indicate the number of cells/electrodes recorded (*n*) as well as the number of animals (*N*) from which these measurements are drawn. Typically, one cell per slice is used for each pharmacological treatment. Statistical testing was done in MATLAB. Comparisons within conditions were made by two-tailed paired Student's *t* test, treatment versus baseline. Comparisons across conditions or between genotypes were done with an unpaired *t* test. For multiple comparisons, a one-way or two-way ANOVA was done with a Bonferroni *post hoc* test. Holm's correction for multiple comparisons has been applied to *p* values (listed as corrected *p*) where WT postnatal time points were reused as age-matched controls for the KN-62 data, or *Elfn1 KO* data were reused in comparison with age-matched controls at P21. Statistical outcomes are represented in figures as follows: n.s., *p* > 0.05, *p < 0.05, **p < 0.01, and ***p < 0.001.

## Results

### L2/3 Pyr→SST synaptic facilitation matures in the second postnatal week

**Figure 1.**
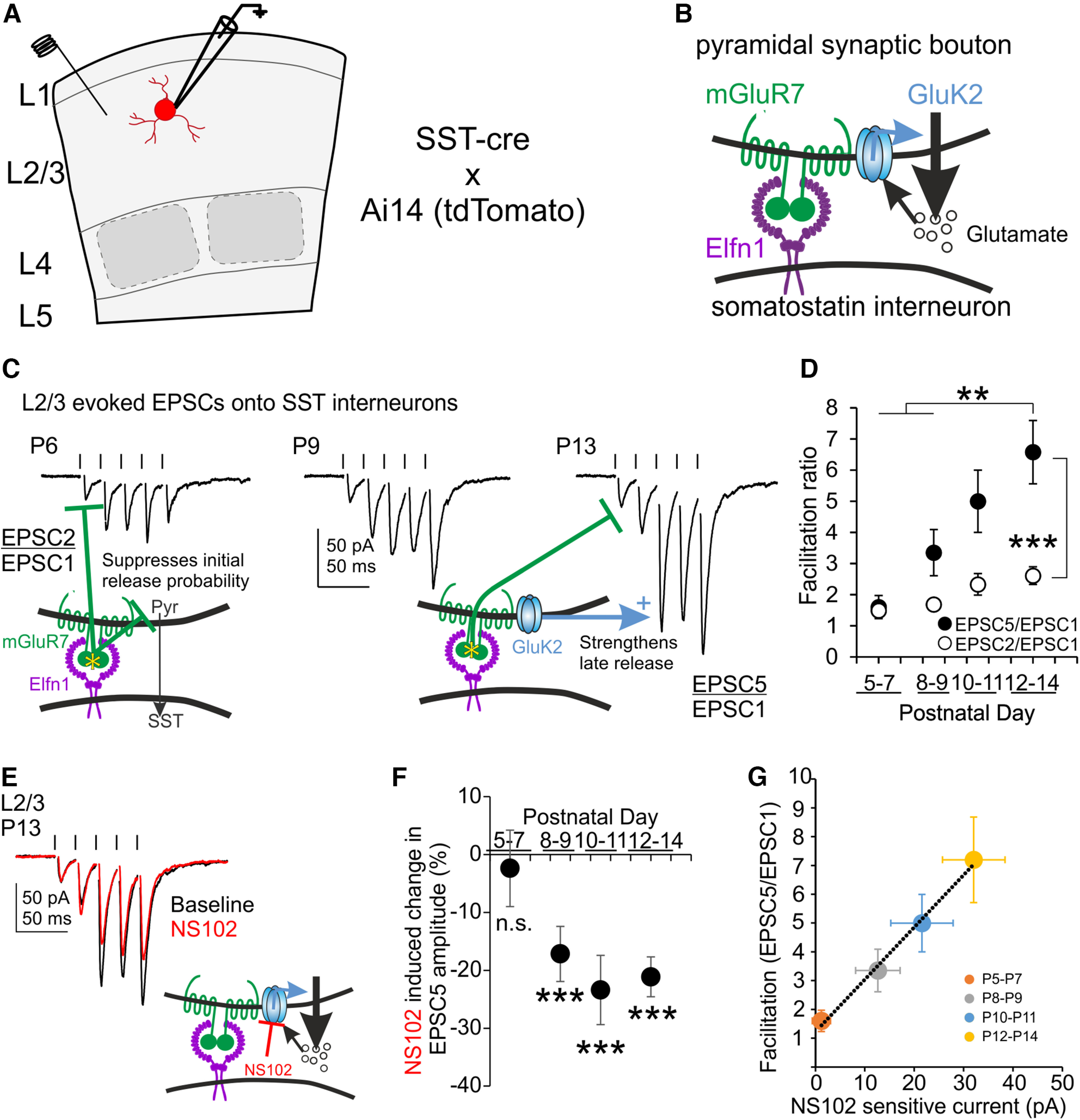
Development of GluK2-KAR responsiveness mirrors increased synaptic facilitation. ***A–E***, Excitatory synaptic transmission onto L2/3 SST synapses is evoked by local electrical stimulation (hash marks in ***C***, ***E***). Synaptic facilitation at L2/3 Pyr→SST synapses is a combination of mGluR7-mediated initial suppression of release and GluK2-KAR-mediated late enhancement of release (***B***, ***C***). Recording from L2/3 SST neurons at P5–P14 reveals a rapid increase in synaptic facilitation over the first two postnatal weeks (***D***). Late facilitation (EPSC5/EPSC1) increases more dramatically than early facilitation (EPSC2/EPSC1). By the end of the second postnatal week, the GluK2 antagonist NS102 inhibits late synaptic release (***E***). ***F***, The onset for presynaptic GluK2-KAR responsiveness at L2/3 Pyr→SST synapses appears around P8–P9 and contributes a stable proportion of late (EPSC5) amplitude into adulthood. ***G***, Developmental increases in late facilitation ratio (EPSC5/EPSC1) correlate with increases in GluK2-KAR-sensitive synaptic current; n.s., *p* > 0.05, *p < 0.05, **p < 0.01, ***p < 0.001.

Recording from L2/3 of barrel cortex in acute brain slices from *SST-cre x Ai14* animals, SST neurons display facilitation of EPSCs evoked by repetitive local electrical stimulation (Fig. 1*A*). The characteristic short-term facilitation of Pyr→SST synapses results from at least two components, a glutamate-independent suppression of early transmission by presynaptic mGluR7 and an enhancement of late transmission by presynaptic GluK2-KARs (Fig. 1*B*). To reveal the developmental timeline in which facilitation is established, we stimulated L2/3 Pyr→SST synapses at 50 Hz across the first 2 postnatal weeks and measured early (EPSC2/EPSC1) and late (EPSC5/EPSC1) facilitation ratios, as a proxy for contributions from mGluR7 and GluK2-KARs, respectively.

We found that late facilitation increased rapidly during the second postnatal week, diverging from early facilitation (age effect, *p* = 0.0002; early vs late facilitation effect, *p* = 0.00,004; age × early vs late facilitation interaction *p* = 0.048, *F* = 7.65, 19.01, 2.76, two-way ANOVA; Fig. 1*C*). In contrast, at the earliest postnatal ages examined, EPSC5/EPSC1 did not differ from EPSC2/EPSC1, (P5–P7, EPSC5/EPSC1 1.6 ± 0.4 vs EPSC2/EPSC1, 1.5 ± 0.2, *p* = 1, Bonferroni *post hoc*, *n*/*N* = 7/6), but increased to mature facilitation levels by the end of the second postnatal week (P12–P14, EPSC5/EPSC1 6.6 ± 1.0 vs 1.6 ± 0.4 at P5–P7, *p* = 0.0001, vs 3.4 ± 0.7 at P8–P9, *p* = 0.005, Bonferroni *post hoc*, *n*/*N* = 12/7, *n*/*N* = 7/6, *n*/*N* = 15/7; Fig. 1*D*). Over the same period, EPSC2/EPSC1 did not change significantly (EPSC2/EPSC1. 2.6 ± 0.3 at P12–P14 vs 1.5 ± 0.2 at P5–P7, *p* = 1, Bonferroni *post hoc*, *n*/*N* = 12/7, *n*/*N* = 7/6). The dramatic increase in late facilitation, with only marginal effects on early facilitation, suggested that presynaptic GluK2-KARs were recruited during the second postnatal week, whereas mGluR7 receptor levels remained largely unchanged.

### The developmental recruitment of L2/3 presynaptic GluK2-KARs occurs at the beginning of the second postnatal week

To directly test the age of postnatal onset for GluK2 signaling, we applied NS102, a selective antagonist of GluK2-KARs (Fig. 1*E*). We found that NS102 reversibly inhibits synaptic transmission at barrel cortex Pyr→SST synapses at P12–P14, primarily affecting EPSCs evoked later in the train (20 μm NS102, P12–P14, EPSC5 amplitude, 144 ± 38 pA in NS102 vs 177 ± 41 pA at baseline, *p* = 0.001, *t* = 5.06, df = 7, paired *t* test, *n*/*N* = 8/4). Furthermore, we revealed that GluK2-KAR activity increases sharply during the second postnatal week (age effect, *p* = 0.007; NS102 effect, *p* = 7 × 10^−11^; age × NS102 interaction, *p* = 0.007, *F* = 4.25, 54.79, 4.25, two-way ANOVA; Fig. 1*F*). In contrast, at P5–P7, we find no discernable effect of NS102 on the late EPSC (normalized change in EPSC5 amplitude, −2.4 ± 6.6% vs baseline, *p* = 1, Bonferroni *post hoc*, *n*/*N* = 7/6). Sampling the effect size of NS102 antagonism across postnatal development, we find a rapid onset of NS102 responsiveness beginning at P8-P9, which stabilizes by the end of the second postnatal week (normalized change in EPSC5 amplitude, P8–P9, −17.2 ± 4.8% vs baseline, *p* = 0.0003; P10–P11, −23.4 ± 6.0% vs baseline, *p* = 0.00,003; P12–P14, −21.1 ± 3.4% versus baseline, *p* = 0.0002, Bonferroni *post hoc*, *n/N* = 15/7, *n/N* = 8/5, *n/N* = 8/4; Fig. 1*F*). The normalized effect size of NS102 blockade trended up from P5–P7 to P8–P9 and was significantly greater at P10–P11 and P12–P14 than at P5–P7 (normalized change in EPSC5 amplitude, P8–P9 vs P5–P7, *p* = 0.06; P10–P11 vs P5–P7, *p* = 0.004; P12–P14 vs P5–P7, *p* = 0.02, Bonferroni *post hoc*, *n/N* = 15/7, *n/N* = 8/5, *n/N* = 8/4). Percentage of blockade by NS102 did not differ between P8–P9 and P10–P11 or P12–P14 (*p* = 1, *p* = 1, Bonferroni *post hoc*), indicating that over development, increases in synaptic facilitation and late EPSC amplitude correspond to a proportional increase in GluK2-KARs contributions. We tested this further by plotting the baseline facilitation ratio across ages over the raw amplitude changes produced by NS102 and found there is a linear relationship between the two (Fig. 1*G*). Facilitation increases at a rate of 0.18 ± 0.01 per pA of NS102-sensitive current (*p* = 0.003, *t* statistic = 18.70, df = 3, *r*^2^ = 0.99, linear regression with lmfit in MATLAB, *n/N* = 7/6, *n/N* = 15/7, *n/N* = 8/5, *n/N* = 8/4 for P5–P7, P8–P9, P10–P11, and P12–P14, respectively). EPSC5/EPSC1 facilitation ratio is estimated to initially be no different from unity when no NS102-sensitive current is present (*y* intercept, 1.2 ± 0.2, *p* = 0.4, *t* statistic = 1.16, df = 3, *n* = 4 time points). However, the contribution of GluK2-KAR to late EPSC amplitude is stable across time (∼20%). Although we suspect that NS102 provides only incomplete blockade of presynaptic KARs at doses selective for GluK2, this stability at later developmental periods nonetheless suggests that presynaptic KARs are not the sole contributors to late facilitation of Pyr→SST synapses. Our later experiments also confirm this but do not further explore the alternative mechanisms. In summary, we find that postnatal maturation of L2/3 Pyr→SST synapses involves the insertion of presynaptic GluK2-KARs during the second postnatal week.

### GluK2-KAR expression at Pyr→SST synapses requires sensory input

**Figure 2.**
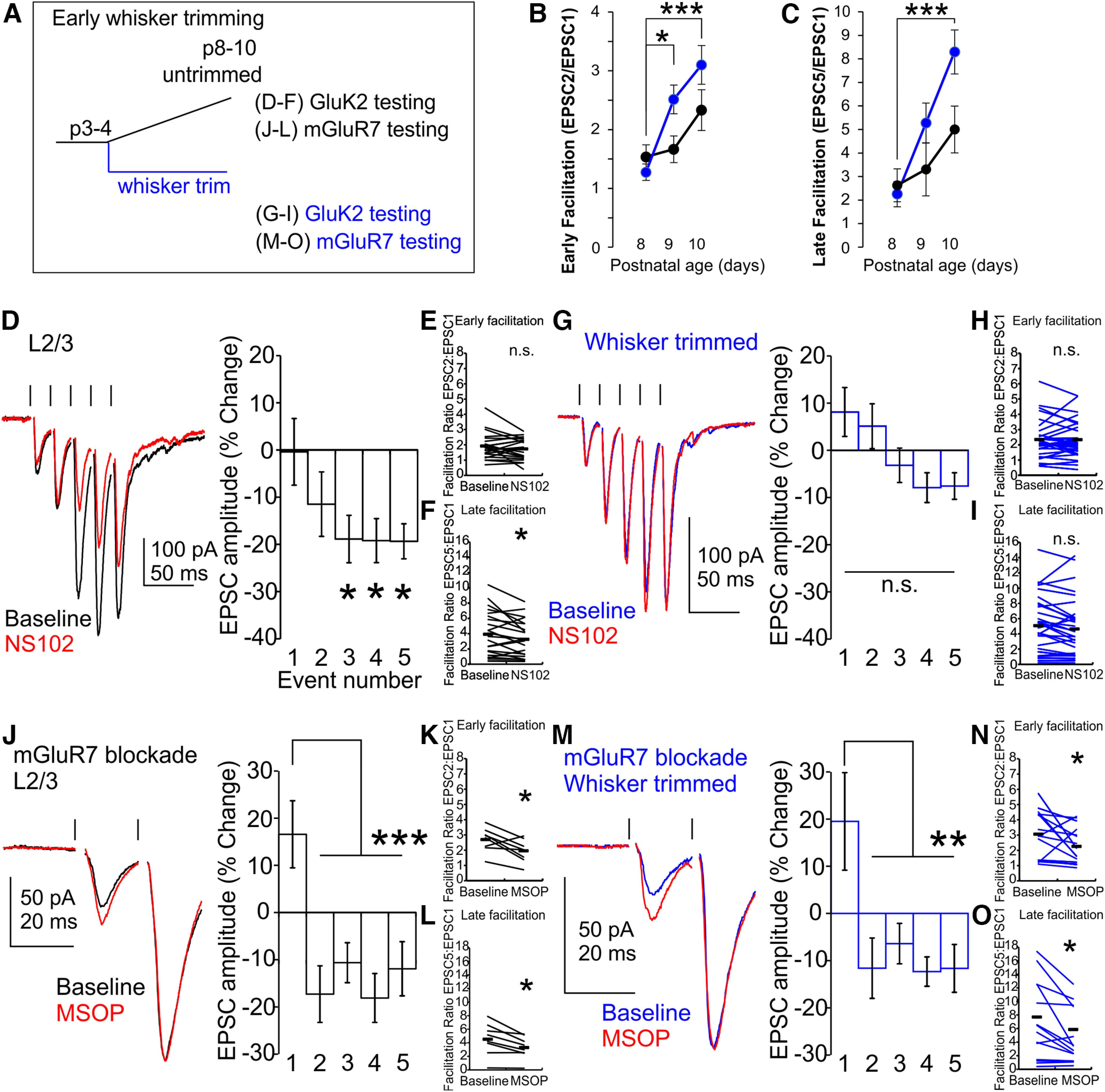
Activity-dependent recruitment of presynaptic GluK2-KARs but not mGluR7 at L2/3 Pyr→SST synapses. ***A***, Experimental outline. Whiskers are trimmed daily (blue). ***B***, ***C***, Whisker trimming increases both early and late facilitation ratio from P8 to P10. In the trimmed condition, early and late facilitation showed a significant increase over development that was not present in the untrimmed group within this timeframe. ***D***, In the untrimmed condition, NS102 reduces late EPSC amplitude in P8–P10 barrel cortex L2/3 SST neurons. ***E***, Early facilitation is not impaired by GluK2 blockade. ***F***, NS102 reduces late facilitation. ***G***, Whisker trimming from P4 to P10 reduces NS102 responsiveness. ***H***, ***I***, Whisker trimming blocks the effect of NS102 on late facilitation. ***J–L***, Whisker trimming does not affect mGluR7 activity at L2/3 Pyr→SST synapses. MSOP increases first pulse amplitude (***J***) and reduces both early (PPR2/1; ***K***) and late facilitation (PPR5/1; ***L***). ***M–O***, In whisker-trimmed cortex, response to MSOP does not differ from untrimmed cortex; n.s., *p* > 0.05, *p < 0.05, **p < 0.01, ***p < 0.001.

In the first 4 postnatal weeks, sensorimotor activity matures rapidly, including the initiation of whisking behavior (Landers and Zeigler, 2006; Arakawa and Erzurumlu, 2015) and the rapid increase and subsequent decline in magnitude of whisker-evoked cortical responses (van der Bourg et al., 2019; Cai et al., 2022). We therefore sought to determine whether tactile sensing by the whiskers regulates the maturation of the barrel cortex Pyr→SST synapses that we observed during this period. It has been shown that whisker trimming reduces the total sensory input to somatosensory cortex without substantial detriment to cortical structure if performed after ∼P4 (Erzurumlu and Gaspar, 2012). Therefore, starting from P4, we trimmed the whiskers of pups daily, continuing through the maturation window for GluK2-KAR upregulation (Fig. 2*A*). Previously it has been shown that in L2/3 barrel cortex, whisker trimming for 4+ days reduces excitatory synaptic strength by reducing release probability, resulting in an increased facilitation ratio (Bender et al., 2006). In accordance, we also found that whisker trimming increased both early and late facilitation at Pyr→SST synapses from P8 to P10, consistent with a reduction in excitatory synaptic strength via decreased initial release probability (early facilitation, P8 trimmed vs P9 trimmed, *p* = 0.04; P8 trimmed vs P10 trimmed, *p* = 0.0006; Fig. 2*B*; late facilitation, P8 trimmed vs P10 trimmed, *p* = 0.0005; Fig. 2*C*, two-way ANOVA with Bonferroni *post hoc*; untrimmed, *n*/*N* = 9/5, 9/5, 8/5; trimmed, *n*/*N*= 9/4, 20/8, 16/7 for P8, P9, and P10, respectively). Early and late facilitation ratios also trended up in untrimmed barrel cortex over this period, and although not significantly increased, it thus remains unclear whether developmental age or length of trimming period or both were affecting release probability. Regardless, these changes confirm that within this time frame, our trimming paradigm reduces Pyr→SST synaptic strength.

We then assessed whether developmental GluK2-KAR expression was dependent on sensory activity. We therefore compared NS102 responsiveness of Pyr→SST synapses in L2/3 barrel cortex from whisker-trimmed animals with those recorded from age-matched untrimmed control cortex. Application of NS102 to slices from control mice suppresses evoked EPSC amplitude for the late events in a 50 Hz stimulus train (P8–P10, normalized change in EPSC in NS102, −18.9 ± 5.1% for EPSC3, −19.2 ± 4.7% for EPSC4, and −19.3 ± 3.7% for EPSC5, *p* = 0.04, *p* = 0.03, and *p* = 0.03, respectively; vs baseline, two-way ANOVA, Bonferroni *post hoc*, *n*/*N* = 23/12; Fig. 2*D*). Correspondingly, GluK2-KAR blockade selectively altered late facilitation without affecting early facilitation (EPSC2/EPSC1 ratio, paired pulse ratio (PPR), 1.7 ± 0.1 in NS102 vs 1.9 ± 0.2 at baseline, *p* = 0.2, *t* statistic = 1.46, paired *t* test, Fig. 2*E*; EPSC5/EPSC1 ratio, 3.3 ± 0.5 in NS102 vs 3.9 ± 0.6 at baseline, *p* = 0.03, *t* statistic = 2.38, paired *t* test; Fig. 2*F*). In contrast, SST cells from whisker-trimmed mouse slices do not display robust NS102 responsiveness (whisker trimmed, P8–P10, normalized change in EPSC in NS102, −6.3 ± 3.4% for EPSC3, −10.7 ± 3.3% for EPSC4, and −8.0 ± 3.0% for EPSC5, *p* = 1, *p* = 0.2, and *p* = 1 vs baseline, respectively; two-way ANOVA, Bonferroni *post hoc*, *n*/*N* = 31/11; Fig. 2*G*). Neither late nor early facilitation was significantly changed by the application of NS102 (EPSC2/EPSC1 ratio, 2.3 ± 0.2 in NS102 vs 2.3 ± 0.2 at baseline, *p* = 0.9, *t* statistic = 0.08, paired *t* test; Fig. 2*H*; EPSC5/EPSC1 ratio, 4.6 ± 0.7 in NS102 vs 5.1 ± 0.7 at baseline, *p* = 0.09, *t* statistic = 1.76, paired *t* test; Fig. 2*I*).

As already stated, the emergence of facilitation at Pyr→SST synapses is because of the coordinated activity of mGluR7 and GluK2-KARs (Stachniak et al., 2019). We therefore tested whether mGluR7 also showed activity-dependent regulation. As previously reported for P11–P19 brain slices (Stachniak et al., 2019), inhibition of constitutive mGluR7 activity with MSOP, a type III mGluR antagonist, increased the first EPSC amplitude in a stimulus train in brain slices from untrimmed animals (P9–P14, normalized change in EPSC1 in MSOP, +16.6 ± 7.1%, *p* = 5 × 10^−6^, 0.0005, 3 × 10^−6^, and 0.0002 vs EPSC2, EPSC3, EPSC4, and EPSC5, respectively; two-way ANOVA, Bonferroni *post hoc*, *n*/*N* = 8/3; Fig. 2*J*). Consequently, both early and late facilitation are reduced (P9–P14 EPSC2/EPSC1 ratio, 2.0 ± 0.3 in MSOP vs 2.7 ± 0.3 at baseline, *p* = 0.02, *t* statistic = 2.90, df = 7, paired *t* test, *n*/*N* = 8/3; Fig. 2*K*; EPSC5/EPSC1 ratio, 3.3 ± 0.6 in MSOP vs 4.5 ± 0.8 at baseline, *p* = 0.01, *t* statistic = 3.37, df = 7, paired *t* test, *n*/*N* = 8/3; Fig. 2*L*). In contrast to the activity-dependent recruitment of GluK2-KARs, however, mGluR7 engagement was not altered by whisker trimming. In whisker-trimmed cortex, MSOP still increased first EPSC amplitude and reduced early and late facilitation to a similar extent as that seen in control mice (P9–P13, normalized change in EPSC1 in MSOP, +19.5 ± 10.3%, *p* = 0.0001, 0.003, 6 × 10^−5^, and 0.0001 vs EPSC2, EPSC3, EPSC4, and EPSC5, respectively; two-way ANOVA, Bonferroni *post hoc*, *p* = 0.9 vs control, *t* statistic = 0.18, df = 23, *t* test, *n/N* = 17/6, *n/N* = 8/3; Fig. 2*M*; EPSC2/EPSC1 ratio, 2.3 ± 0.3 in MSOP vs 3.1 ± 0.4 at baseline, *p* = 0.04, *t* statistic = 2.26, df = 16, paired *t* test, *n*/*N* = 17/6; Fig. 2*N*; EPSC5/EPSC1 ratio, 5.9 ± 1.1 in MSOP vs 7.7 ± 1.3 at baseline, *p* = 0.02, *t* statistic = 2.56, df = 16, paired *t* test, *n*/*N* = 17/6; Fig. 2*O*). Therefore, our experimental results did not detect changes in mGluR7 activity that could account for the increases in early and late facilitation induced by whisker trimming. This could instead be attributed to an LTD-like mechanism (Bender et al., 2006), which we do not explore further here. Thus, we find that whisker-dependent cortical activation is necessary for GluK2-KAR recruitment to Pyr→SST synapses but not for early facilitation via mGluR7.

### Whisker regrowth or calmodulin/CaMKII activation reinstates GluK2-KARs

**Figure 3.**
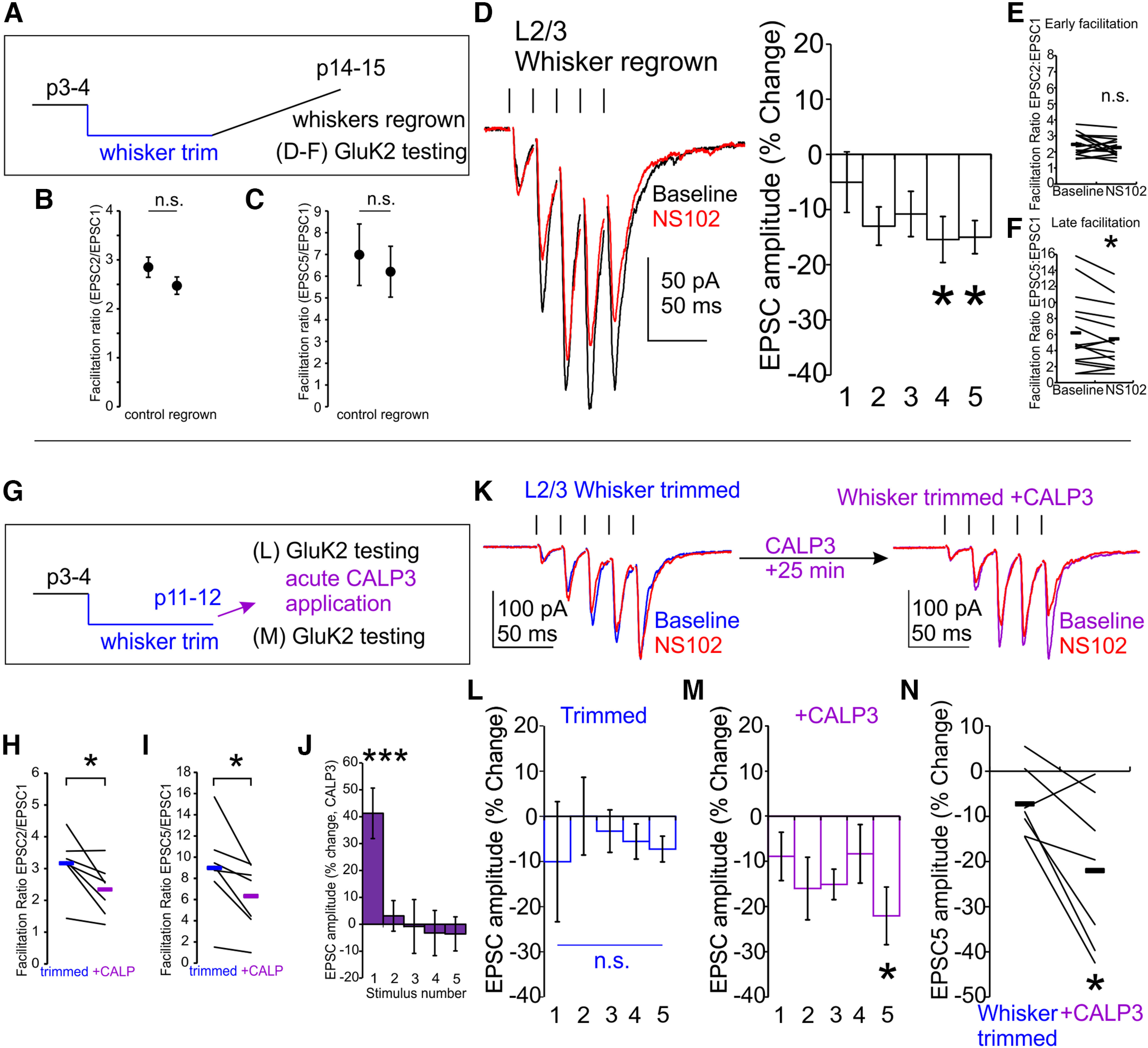
Reversal of the loss of GluK2-KARs by restoration of activity or calmodulin activation. ***A***, Following whisker trimming (P4–P10), whiskers are allowed to regrow. ***B***, ***C***, In cortex from animals with whiskers regrown, early and late facilitation ratios do not differ from control. ***D–F***, Whisker regrowth after trimming restores NS102 responsiveness and restores effects on early and late facilitation to normal. ***G***, Calmodulin activation recruits presynaptic GluK2-KAR expression at L2/3 barrel cortex Pyr→SST synapses. ***H***, ***I***, CALP3 treatment decreases early and late facilitation ratio at L2/3 Pyr→SST synapses from whisker-trimmed mice. ***J***, These changes are associated with increased first EPSC amplitude. ***K***, Calmodulin activation with CALP3 induces changes in Pyr→SST synapses that stabilize after ∼25 min. ***L***, In whisker-trimmed animals, at L2/3 Pyr→SST synapses are not NS102 responsive (blue). ***M***, Application of the calmodulin activator CALP3 induces responsiveness to NS102 (magenta). ***N***, Pairwise comparison before and after CALP3 treatment shows a significant increase in NS102-induced changes in EPSC5 amplitude; n.s., *p* > 0.05, *p < 0.05.

To determine whether the failure to recruit GluK2-KAR to Pyr→SST synapses in whisker-trimmed cortex was reversible or rather a purely developmental phenomenon, we allowed whiskers to regrow and tested NS102 responsiveness again (Fig. 3*A*). Following whisker regrowth, early and late facilitation ratios did not differ from control conditions (EPSC2/EPSC1 ratio, *p* = 0.3, *t* statistic = 1.39, df = 25, *t* test; Fig. 3*B*; EPSC5/EPSC1 ratio, *p* = 0.5, *t* statistic = 0.43, df = 25, *t* test, *n*/*N* = 15/5, *n*/*N* = 12/6 for regrown and P14–P15 control, respectively; Fig. 3*C*). NS102 responsiveness also resembled control conditions, indicating that maturation deficits at Pyr→SST synapses are reversible (whisker trimmed, regrown P14–P15, normalized change in EPSC in NS102, −15.4 ± 4.1% for EPSC4 and −15.0 ± 3.9% for EPSC5, *p* = 0.01 and *p* = 0.02, respectively, vs baseline, two-way ANOVA, Bonferroni *post hoc*; Fig. 3*D*; EPSC2/EPSC1 ratio, 2.3 ± 0.1 in NS102 vs 2.5 ± 0.2 at baseline, *p* = 0.3, *t* statistic = 1.19, df = 14, paired *t* test, *n*/*N* = 15/5; Fig. 3*E*; EPSC5/EPSC1 ratio, 5.5 ± 1.0 in NS102 vs 6.2 ± 1.2 at baseline, *p* = 0.03, *t* statistic = 2.46, df = 14, paired *t* test, *n*/*N* = 15/5; Fig. 3*F*).

The re-insertion of GluK2-KAR at Pyr→SST synapses following regrowth of whiskers after trimming shows that sensory-evoked activity in barrel cortex can dynamically regulate GluK2-KARs. To determine whether the GluK2 recruitment could be activated acutely, we examined the signaling cascades involved in the dynamic regulation of GluK2-KARs using pharmacology in slices (Fig. 3*G*). By measuring acute changes in GluK2-KARs, we also rule out the effects of any structural changes that whisker trimming might evoke. Activity-dependent changes in postsynaptic KAR surface expression, motility, and synaptic localization have been attributed to calmodulin/CaMKII signaling cascades (Carta et al., 2013). We find that acute activation of calmodulin with a brief application of calcium-like peptide 3 (CALP3; 100 μm; a synthetic calmodulin activator) increases initial synaptic release, reducing early and late facilitation ratios, as one might see with LTP induction (CALP3 effect, early facilitation, *p* = 0.02, *t* = 3.08; Fig. 3*H*; late facilitation, *p* = 0.02, *t* = 3.08; Fig. 3*I*; df = 6, paired *t* test, *n*/*N* = 7/3; ESPC1 amplitude, CALP3 effect, *p* = 3 × 10^−7^; Fig. 3*J*; two-way ANOVA with Bonferroni *post hoc*). CALP3 is a cell-permeable peptide, and this effect emerges slowly after CALP3 application (25+ min after a 3-min-long application of CALP3).

Having shown that CALP3 enhances synaptic transmission at Pyr→SST synapses, we then tested the effect on presynaptic GluK2-KARs. We find that CALP3 induces the recruitment of GluK2-KARs to synapses from whisker-trimmed mice (Fig. 3*K*). L2/3 SST neurons that were initially unresponsive to NS102 showed increased responsiveness after the acute treatment with CALP3 (P11–P12 whisker trimmed, normalized change in EPSC5 in NS102, −22.0 ± 6.4% post-CALP3 vs −7.3 ± 2.9% at baseline, *p* = 0.03, *t* statistic = 2.88, paired *t* test, *n*/*N* = 7/3; Fig. 3*L–N*). Thus, the effects of whisker trimming are reversed as the whiskers are restored, with GluK2-KARs rapidly recruited to the synapse in response to calmodulin activation.

### Late whisker trimming or calmodulin/CaMKII blockade removes GluK2-KARs

**Figure 4.**
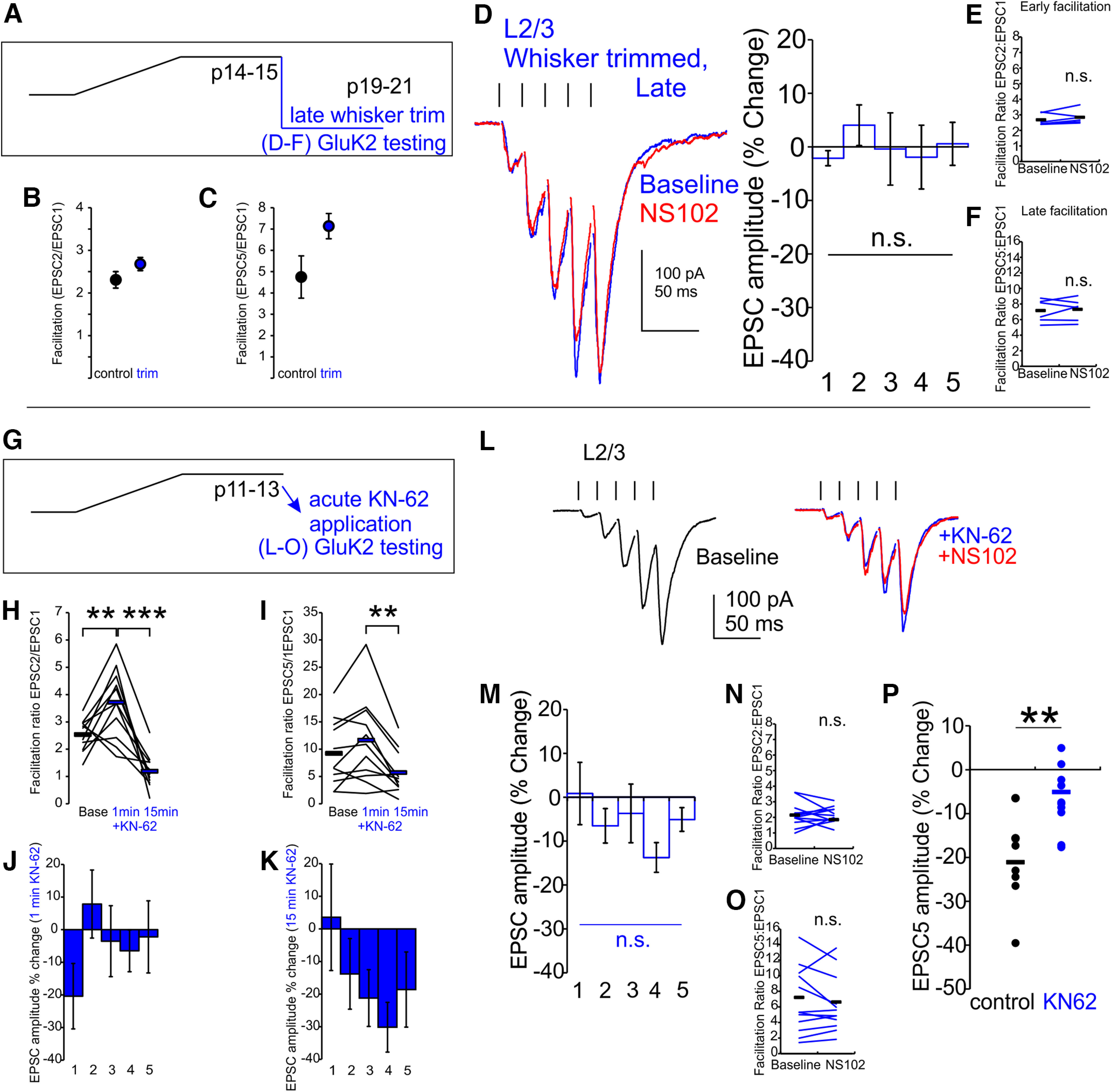
CaMKII signaling regulates presynaptic GluK2-KAR maintenance at L2/3 Pyr→SST synapses. ***A***, Trimming is performed in late development (P14–P21), after GluK2-KARs have already been recruited. ***B***, ***C***, Late whisker trimming does not significantly increase the early or late facilitation ratio compared with control, although an upward trend is found. ***D–F***, Late trimming still reduces GluK2 activity back to negligible levels. Late whisker trimming also blocks NS102 effects on late facilitation. ***G***, The CaMKII inhibitor KN-62 is applied to normal cortex. ***H–K***, Application of KN-62 changes early and late facilitation ratios. In the first minute of KN-62 application, facilitation rapidly increases, associated with changes in first EPSC amplitude. By 15 min later, facilitation is reduced, associated with reductions in later event amplitude. ***L***, KN-62 reduced facilitation slightly, and subsequent application of NS102 failed to inhibit evoked EPSC amplitude (blue). ***M***, KN-62 treatment reduces responsiveness to NS102. ***N–O***, Early and late facilitation does not respond to NS102 in KN-62-treated slices. ***P***, NS102-induced change in EPSC5 amplitude is reduced in KN-62-treated slices (blue) compared with age matched control slices (black, replotted from Fig. 1*F*); n.s., *p* > 0.05, *p < 0.05, **p < 0.01.

The activity-dependent reinstatement of KARs after whisker trimming suggests a persistent flexibility of Pyr→SST synapse composition. Conversely, synaptic remodeling in L2/3 conforms to strict critical periods (P12–P16) for experience-dependent plasticity, outside of which, whisker deprivation no longer triggers remodeling (Wen and Barth, 2011). Likewise, activity-dependent maturation of the intrinsic electrophysiological properties of SST cells is sensitive to the disruption of Pyr→SST synaptic activity in early but not later developmental periods (Pan et al., 2019). We therefore tested whether whisker trimming would still produce reductions in GluK2-KAR engagement, after the Pyr→SST synapses had reached a mature state (Fig. 4*A*). Late whisker trimming did not significantly increase the early and late facilitation ratios compared with age-matched controls, although a trend toward an increase was seen (EPSC2/EPSC1 ratio, *p* = 0.2, *t* statistic = 1.39, df = 14; Fig. 4*B*; EPSC5/EPSC1 ratio, *p* = 0.09, *t* statistic = 1.82, df = 14, *t* test, *n*/*N* = 6/3, *n/N* = 10/3 for whisker trimmed and P21 control, respectively; Fig. 4*C*). In response to NS102, animals with whiskers trimmed after the critical window again showed no change in amplitude or facilitation ratio (whisker trimmed P19–P21, normalized change in EPSC5 in NS102, +0.6 ± 4.0%, *p* = 1 vs baseline, two-way ANOVA, Bonferroni *post hoc*, *n*/*N* = 6/3; Fig. 4*D*; EPSC2/EPSC1 ratio, 2.8 ± 0.2 in NS102 vs 2.7 ± 0.2 at baseline, *p* = 0.2, *t* statistic = 1.46, df = 5, paired *t* test, *n/N* = 6/3; Fig. 4*E*; EPSC5/EPSC1 ratio, 7.3 ± 0.6 in NS102 vs 7.1 ± 0.6 at baseline, *p* = 0.6, *t* statistic = 0.50, df = 5, paired *t* test, *n/N* = 6/3; Fig. 4*F*).

The activity-dependent insertion or removal of GluK2-KAR from synapses can help explain how whisker trimming alters NS102 sensitivity, even after the critical window for activity-induced plasticity of sensory cortex has elapsed. As late whisker trimming is sufficient to downregulate GluK2-KARs, we hypothesized that maintenance of presynaptic GluK2-KAR expression also uses an activity-dependent cascade. We therefore tested whether GluK2-KAR expression requires ongoing maintenance of calmodulin signaling even after postnatal maturation by applying the CaMKII antagonist KN-62 to acute cortical slices from control mice (Fig. 4*G*). CaMKII signaling cascades are involved in a multitude of processes, and, accordingly, the rapid onset of KN-62 allowed us to see a biphasic effect on early and late facilitation. Initially, a rapid decrease in synaptic release (first minute of application) produced an increase in both early and late facilitation (KN-62 effect, early facilitation, *p* = 0.008; 1 min vs baseline, *p* = 0.0006; 15 min vs 1 min, *p* = 1; 15 min vs baseline; late facilitation, *p* = 0.2; 1 min vs baseline, *p* = 0.003; 15 min vs 1 min, *p* = 0.2; 15 min vs baseline, *n*/*N* = 11/6, ANOVA with Bonferroni *post hoc*; Fig. 4*H–J*). At stable state (15+ min), a drop in facilitation resulted from changes in later events, qualitatively similar to what might be expected from a loss of GluK2-KARs (Fig. 4*K*), normalizing facilitation ratio.

As predicted from the late trimming experiments, acute treatment with KN-62 resulted in a blunted responsiveness to NS102 for amplitude and facilitation (KN-62-treated P13–P16 normalized change in EPSC in NS102, −13.8 ± 3.4% for EPSC4 and −5.1 ± 2.7% for EPSC5, *p* = 0.4 and *p* = 1, respectively, vs baseline, two-way ANOVA, Bonferroni *post hoc*, *n*/*N* = 11/6; Fig. 4*L*,*M*; EPSC2/EPSC1 ratio, 2.1 ± 0.2 in NS102 vs 2.3 ± 0.2 at baseline, *p* = 0.4, *t* statistic = 0.83, df = 10; Fig. 4*N*; EPSC5/EPSC1 ratio, 6.6 ± 1.1 in NS102 vs 7.2 ± 1.2 at baseline, *p* = 0.4, *t* statistic = 0.97, df = 10, paired *t* test, *n*/*N* = 11/6; Fig. 4*O*). When comparing to age-matched control slices, NS102 responsiveness was reduced by KN-62 application (P12–P14, normalized change in EPSC5 in NS102, −5.1 ± 2.7% KN-62-treated vs −21.1 ± 3.4% control slices, corrected *p* = 0.007, *t* statistic = 3.71, *t* test, *n*/*N* =11/6, *n*/*N* = 8/4; Fig. 4*P*).

Although CALP3 and KN-62 application altered responsiveness to NS102, the effects on the facilitation ratio were complex. Substantial changes in initial synaptic release occluded any changes in the facilitation ratio that were expected to accompany the changes in GluK2-KAR expression. This dissociation of GluK2-KAR activity from the facilitation ratio during pharmacological or whisker manipulations could thus be because of confounding effects of both presynaptic and postsynaptic changes, which are induced by calmodulin/CaMKII or other activity manipulations, including effects on initial synaptic release probability (Xia and Storm, 2005; Bender et al., 2006).

In summary, we find that presynaptic GluK2-KARs are recruited and maintained at Pyr→SST synapses through an activity-dependent cascade that includes calmodulin/CaMKII signaling. Additionally, activity-dependent regulation of presynaptic GluK2-KARs is not restricted to the classical critical developmental window but rather is initiated at the beginning of the second postnatal week and thereafter is available on demand. Conversely, neither mGluR7 activity nor the emergence of facilitation were impaired by the loss of sensory activity following whisker trimming.

### Developmental shift in layer-specific presynaptic KARs at Pyr→SST synapses

**Figure 5.**
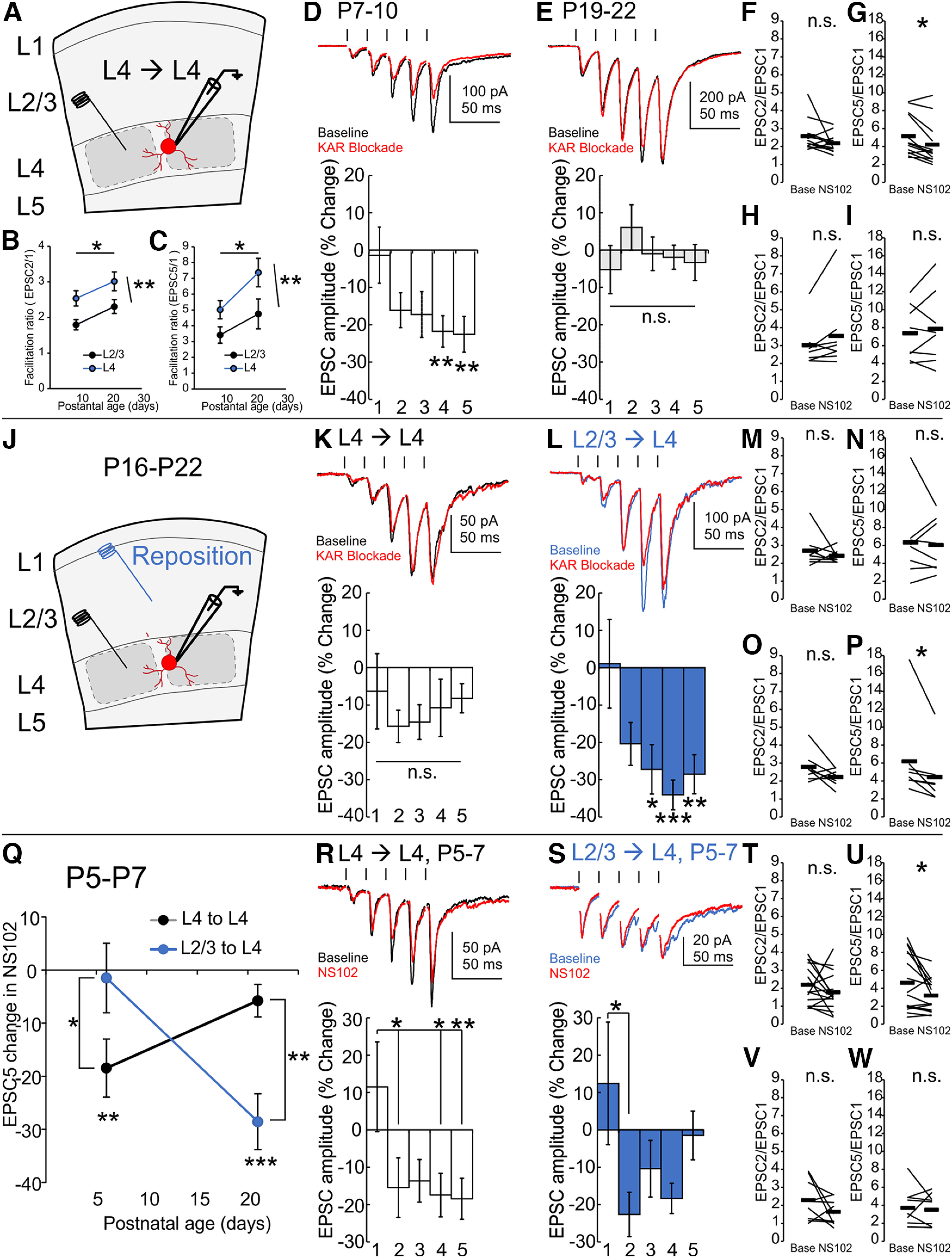
Developmental switch in locus of sensory perception from bottom-up to top-down control. Activity-dependent induction or loss of presynaptic GluK2-KAR expressions tracks the repositioning of sensory perception over the first 2 postnatal weeks. ***A***, Stimulation (left electrode) within L4 evokes KAR-dependent facilitation on L4 SST interneurons (right electrode) at P7. ***B***, ***C***, Development increases early and late facilitation ratios in L2/3 and L4. L4 facilitates more than L2/3. ***D***, KAR blockade produced significant inhibition of late event amplitude in L4 Pyr→L4 SST synapses at P7–P10. ***E***, The transition to active whisking increases reliance on central resources and thus reduces bottom-up inhibition. KAR blockade has no effect at L4 to L4 Pyr→SST synapses at P21. ***F***, ***G***, At P7-P10, KAR blockade reduces late but not early facilitation at L4 Pyr→L4 SST synapses. ***H***, ***I***, At P21, facilitation is not affected at L4 Pyr→L4 SST synapses. ***J***, At P21, L4 SST cells show differential sensitivity to KAR blockade for L2/3 stimulation than for L4 stimulation. ***K***, ***L***, Pairs of recordings from stimulation from either L4 Pyr or L2/3 Pyr→L4 SST cells showed substantially greater inhibition in response to KAR blockade from L2/3 at P16–P22. ***M***, ***N***, At P21, facilitation is not affected at L4 Pyr→L4 SST synapses. ***O***, ***P***, In the same SST cells, KAR blockade reduces late but not early facilitation at L2/3 Pyr→L4 SST synapses. ***Q***, At P5–P7, L2/3 Pyr→L4 SST synapses did not respond to NS102, as measured by EPSC5 inhibition. L4 Pyr→L4 SST synapses show significantly larger responses. The inverse response profile was present at P21 (data from ***C***, ***I***, ***J***). ***R***, ***S***, NS102 produces inhibition of late event amplitude at L4 Pyr→L4 SST synapses but not at L2/3 Pyr→L4. ***T***–***W***) Late but not early synaptic facilitation is selectively affected at L4 to L4 at P5–P7; n.s., *p* > 0.05, *p < 0.05, **p < 0.01, ***p < 0.001.

The requirement for activity and calmodulin/CaMKII signaling to maintain presynaptic GluK2-KARs at L2/3 Pyr→SST synapses predicts that underused synapses might undergo elimination of presynaptic GluK2-KAR. We therefore examined L4 Pyr→L4 SST synapses at two developmental time points, at P7–P10, when passive whisker deflection dominates whisker sensation, and at P21, when active whisking is well established. We recorded excitatory synaptic responses from L4 barrel cortex SST interneurons while repetitively stimulating within L4 (Fig. 5*A*). L4 Pyr→L4 SST synapses generally showed more facilitation than in L2/3 (ANOVA main effect for cortical layer, early facilitation, *p* = 0.004, *F* = 9.12; Fig. 5*B*; late facilitation, *p* = 0.009, *F* = 7.32; Fig. 5*C*). From P7–P10 to P21, both early and late facilitation ratios increased in L4 and also in L2/3, confirming that over development, the facilitation ratio increases through a GluK2-independent reduction of initial release strength (ANOVA main effect for development, early facilitation, *p* = 0.04, *F* = 4.25; late facilitation, *p* = 0.02, *F* = 5.6, two-way ANOVA, *n*/*N* = 18/7, 8/6, 29/13, 10/3, respectively, for L4, P7–P10, P21 and L2/3, P7–P10, P21).

We then theorized that independent of global developmental changes in synaptic release outlined above, the extensive use of top-down control of whisker-driven sensory responses at P21 necessitates GluK2-KAR engagement in L2/3. Conversely, L4 is strongly engaged earlier in development but is used less as barrel cortex matures (Cai et al., 2022), perhaps prompting GluK2-KAR elimination from L4 Pyr→L4 SST synapses. To test this theory, we blocked presynaptic KARs and find that they are present at these synapses at P7–P10 (P7–P10, normalized change in EPSC in 10 μm UBP310, −21.8 ± 4.1% for EPSC4 and −22.5 ± 4.8% for EPSC5, *p* = 0.006 and *p* = 0.003, respectively, vs baseline, two-way ANOVA, Bonferroni *post hoc*, *n*/*N* = 13/5; Fig. 5*D*). Further, GluK2-KARs persist in L4 at P12 (P10–P13, normalized change in EPSC in 20 μm NS102, −15.4 ± 5.5% for EPSC3, −17.2 ± 4.8% for EPSC4, and −15.6 ± 3.2% for EPSC5, *p* = 0.04, *p* = 0.01, and *p* = 0.03, respectively; vs baseline, two-way ANOVA, Bonferroni *post hoc*, *n*/*N* = 10/6). By P21, however, L4 barrel cortex Pyr→SST synapses do not show substantial engagement of KAR activity (P19–P22, normalized change in EPSC in NS102/UBP310, −1.9 ± 3.2% for EPSC4 and −3.3 ± 4.8% for EPSC5, *p* = 1, *p* = 1, respectively; vs baseline, two-way ANOVA, Bonferroni *post hoc*, *n*/*N* = 8/6; Fig. 5*E*). As with L2/3, KAR blockade does not reduce early facilitation but reduces late facilitation for L4 Pyr→L4 SST synapses, at P7–P10 (EPSC2/EPSC1 ratio, 2.2 ± 0.1 in NS102 vs 2.6 ± 0.2 at baseline, *p* = 0.1, *t* statistic = 1.63, df = 12, paired *t* test, *n*/*N* = 13/5; Fig. 5*F*; EPSC5/EPSC1 ratio, 4.2 ± 0.7 in NS102 vs 5.1 ± 0.6 at baseline, *p* = 0.005, *t* statistic = 3.44, df = 7, paired *t* test; Fig. 5*G*), which is lost at P19–P22 (EPSC2/EPSC1 ratio, 3.5 ± 0.7 in NS102 vs 3.0 ± 0.4 at baseline, *p* = 0.1, *t* statistic = 1.76, df = 12, paired *t* test, *n*/*N* = 8/6; Fig. 5*H*; EPSC5/EPSC1 ratio, 7.9 ± 1.4 in NS102 vs 7.3 ± 1.1 at baseline, *p* = 0.5, *t* statistic = 0.74, df = 7, paired *t* test; Fig. 5*I*). Elevated KAR expression at early postnatal ages is consistent with the proposition that passive whisker activation engages the barrel circuit primarily via bottom-up input into L4 at these ages, whereas actively whisking mice (P21) show minimal KAR activity in L4.

As the cortex matures there is a reduced reliance on purely bottom-up feedforward sensation. We hypothesized that the top-down control of whisking could be used to influence sensory processing via projections from L2/3 to L4 (Hooks et al., 2011). Projection-specific modulation of presynaptic GluK2-KAR content could allow for differences between L2/3 Pyr→L4 SST and L4 Pyr→L4 SST synapses, even onto the same cell (Fig. 5*J*). At P21, EPSCs evoked from L4 onto the same L4 SST cells were not sensitive to KAR blockade (normalized reduction in EPSC in UBP310/NS102, P16–P22, 14.6 ± 4.6% for EPSC3, 10.8 ± 7.7% for EPSC4, and 8.2 ± 3.9% for EPSC5, *p* = 1, *p* = 1, *p* = 1, respectively; vs baseline, two-way ANOVA, Bonferroni *post hoc*, *n*/*N* = 8/6; Fig. 5*K*). In contrast, L2/3 stimulation evoked facilitating EPSCs onto L4 SST interneurons that could be attenuated by KAR blockade with NS102 or UBP310 (normalized reduction in EPSC in UBP310/NS102, P16–P22, 27.3 ± 6.6% for EPSC3, 34.1 ± 4.0% for EPSC4, and 28.6 ± 5.3% for EPSC5, *p* = 0.02, *p* = 0.0005, *p* = 0.008, respectively; vs baseline, two-way ANOVA, Bonferroni *post hoc*, *n*/*N* = 8/6; Fig. 5*L*). In these matched pairs of recordings, facilitation was not affected by KAR blockade at L4 Pyr→L4 SST synapses (EPSC2/EPSC1 ratio, 2.4 ± 0.1 in NS102 vs 2.7 ± 0.3 at baseline, *p* = 0.4, *t* statistic = 0.82, df = 7, paired *t* test, *n*/*N* = 8/6; Fig. 5*M*; EPSC5/EPSC1 ratio, 6.0 ± 1.0 in NS102 vs 6.3 ± 1.5 at baseline, *p* = 0.7, *t* statistic = 0.35, df = 7, paired *t* test; Fig. 5*N*), but late facilitation was impaired when switching to stimulation from L2/3 Pyr→L4 SST synapses (EPSC2/EPSC1 ratio, 2.2 ± 0.2 in NS102 vs 2.8 ± 0.3 at baseline, *p* = 0.1, *t* statistic = 1.93, df = 7, paired *t* test, *n*/*N* = 8/6, Fig. 5*O*; EPSC5/EPSC1 ratio, 4.4 ± 1.1 in NS102 vs 6.2 ± 1.6 at baseline, *p* = 0.04, *t* statistic = 2.53, df = 7, paired *t* test; Fig. 5*P*). These findings not only demonstrate that presynaptic GluK2 regulation is not determined postsynaptically and thus can be projection specific but also suggest that feedback to L4 through a L2/3 Pyr→L4 SST pathway could enable the use of top-down information by L4 SST cells in barrel cortex.

We tested this hypothesis by comparing NS102-sensitive responses in L4 seen at P21 with responses at P5–P7 (Fig. 5*Q*), before the maturation of L2/3 Pyr→L2/3 SST synapses. Although not as robust as at P7–P10, L4 to L4 Pyr→SST synapses nonetheless show NS102 responsiveness at P5–P7 (P5–P7, normalized change in EPSC in NS102, −17.5 ± 5.8% for EPSC4, and −18.5 ± 5.4% for EPSC5, *p* = 0.01, *p* = 0.008, respectively; vs EPSC1, two-way ANOVA, Bonferroni *post hoc*, *n*/*N* = 16/8; Fig. 5*R*). Late but not early facilitation is reduced (EPSC2/EPSC1 ratio, 1.8 ± 0.3 in NS102 vs 2.2 ± 0.3 at baseline, *p* = 0.2, *t* statistic = 1.36, df = 15, paired *t* test, *n*/*N* = 16/8; Fig. 5*S*; EPSC5/EPSC1 ratio, 3.2 ± 0.5 in NS102 vs 4.6 ± 0.8 at baseline, *p* = 0.02, *t* statistic = 2.65, df = 15, paired *t* test; Fig. 5*T*). We then examined GluK2 activity at L2/3 to L4 synapses early in development (Fig. 5*U*). In an inversion of our observations at P21, L2/3 Pyr→L4 SST synapses do not show GluK2-KAR activity at P5–P7 (P5–P7, normalized change in EPSC in NS102, −18.4 ± 4.0% for EPSC4 and −1.5 ± 6.5% for EPSC5, *p* = 0.054, *p* = 1, respectively; vs EPSC1, two-way ANOVA, Bonferroni *post hoc*, *n*/*N* = 9/6; Fig. 5*V*). Facilitation is not affected (EPSC2/EPSC1 ratio, 1.6 ± 0.2 in NS102 vs 2.3 ± 0.4 at baseline, *p* = 0.1, *t* statistic = 1.59, df = 8, paired *t* test, *n*/*N* = 9/6; Fig. 5*T*; EPSC5/EPSC1 ratio, 3.5 ± 0.6 in NS102 vs 3.7 ± 0.7 at baseline, *p* = 0.7, *t* statistic = 0.39, df = 8, paired *t* test; Fig. 5*W*). For both L2/3 and L4 stimulation, we find that these early time points show a trend toward increased EPSC1 amplitude in the presence of NS102, which although not significant, represents a caveat for these experiments (P5–P7, normalized change in EPSC1 in NS102, +11.5 ± 12.0% for L4 to L4 and +12.4 ± 16.4% for L2/3 to L4, *p* = 0.03, *p* = 0.01, respectively, vs EPSC2, two-way ANOVA, Bonferroni *post hoc*, *n*/*N* = 16/8, *n*/*N* = 9/6, respectively; Fig. 5*R*,*S*). We hypothesize that this change could be attributable to presynaptic GluK1-KARs, which are inhibitory at P3–P6, but undergo activity-dependent downregulation and elimination (Lauri et al., 2006). Nonetheless, our results show that presynaptic GluK2-KAR expression switches between L4 at early ages, to L2/3 by P21 (normalized change in EPSC5 in NS102 at P5–P7, −18.5 ± 5.5% in L4 to L4 vs −1.5 ± 6.5% in L2/3 to L4, *p* = 0.04, and *p* = 0.002 vs baseline; P16–P22: −28.5 ± 5.3% in L2/3 to L4 vs −5.8 ± 3.1% in L4 to L4, *p* = 0.002, and *p* = 0.0004 vs baseline, two-way ANOVA, Bonferroni *post hoc*, Fig. 5*Q*).

Because of COVID-19 pandemic-related supply restrictions, a subset of the cells in these experiments used the GluK1/3 antagonist UBP310 (10 μm) instead of NS102. Notably, similar inhibitory profiles were observed for 10 μm UBP310 as for 20 μm NS102 at Pyr→SST synapses (normalized change in EPSC5, +1.2 ± 8.6% for UBP310 vs −7.8 ± 4.6% for NS102, *p* = 0.4, *t* statistic = 0.93, df = 6, *t* test, *n*/*N* = 4/3, 4/3). Statistical outcomes did not change when including or excluding the UBP310 data from the NS102 datasets. As UBP310 has previously shown efficacy against GluK2-containing heteromers, but is ineffective against R-edited GluK2 homodimers or GluK2(R)/GluK3 heterodimers, we interpret our findings to suggest that the presynaptic GluK2-KARs resident at Pyr→SST synapses are unedited GluK2(Q)/GluK3 heterodimers, consistent with their role as calcium-permeable receptors that can increase presynaptic release probability (Perrais et al., 2009; Pinheiro et al., 2013).

### Absence of Pyr→SST synaptic facilitation reduces adaptation of late evoked responses to repetitive whisker stimulation

**Figure 6.**
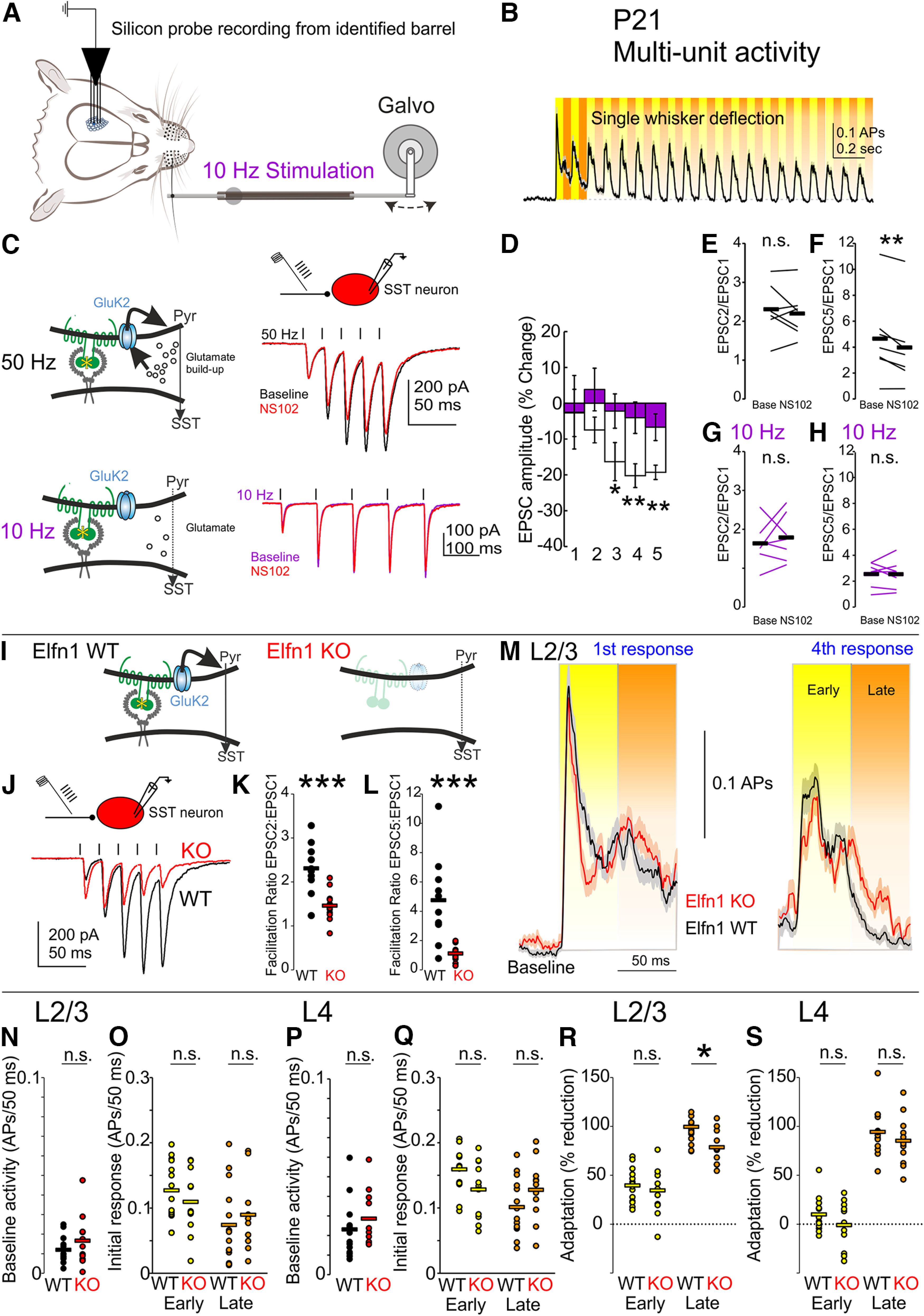
GluK2-KAR removal via *Elfn1 KO* impairs late adaptation. ***A***, Silicon probe L2/3 multiunit activity from identified barrels at P21 in response to repetitive 10 Hz single-whisker deflection. ***B***, Sensory adaptation is calculated as the reduction from the average initial responses (solid orange and yellow bars) of the subsequent responses (shaded orange and yellow bars) over the remaining 18 deflections for early activity (yellow, 0–50 ms) or late activity (orange, 50–100 ms). ***C***, NS102 blockade reduces late amplitude at 50 Hz but is ineffective at 10 Hz stimulation *in vitro*. ***D***, GluK2-KARs are present at L2/3 Pyr→SST synapses at P21 but are not substantially recruited at 10 Hz stimulation, rendering NS102 ineffective. ***E***, ***F***, At 50 Hz, KAR blockade reduces late but not early facilitation. ***G***, ***H***, At 10 Hz, facilitation is not affected at L2/3 Pyr→SST synapses. ***I***, ***J***, Elfn1 contributes to short-term facilitation in Pyr→SST synapses. In the absence of Elfn1, presynaptic mGluR7 and GluK2-KAR are absent, preventing the synaptic facilitation that produces delayed inhibition. ***K***, Early facilitation is reduced as mGluR7 is lost, increasing first EPSC amplitude. ***L***, Late facilitation is reduced without GluK2-KAR to sustain late EPSC amplitude. ***M***, Silicon probe L2/3 multiunit activity is quantified by dividing responses into early activity and late activity (yellow and orange, shaded) allowing some separation of feedforward sensory responses from recurrent activity for initial response to whisker deflection (left). Right, As illustrated in an example response (fourth pulse), attenuation of early and late responses diverges in *Elfn1 KO*. A return to baseline represents 100% attenuation. Traces are mean ± SEM. ***N***, Baseline activity in L2/3 barrel cortex does not differ between WT and KO. ***O***, Initial responses in L2/3 to whisker touch do not differ between WT and KO. ***P***, Baseline activity in L4. ***Q***, Initial responses in L4. ***R***, In *Elfn1 KO*, the absence of late-phase inhibition results in a reduction of late adaptation in L2/3, whereas early adaptation is unaffected. ***S***, In L4, however, neither early- nor late-phase activity shows significant reductions in adaptation in *Elfn1 KO*; n.s., *p* > 0.05, *p < 0.05, ***p < 0.001.

Finally, we sought to assess how sensory-driven cortical responses are influenced by short-term facilitation of excitation onto SST interneurons. We recorded responses to repetitive single-whisker stimulation (10 Hz, 2 s) from identified barrels using multichannel silicon probes in anesthetized P21 mice (Fig. 6*A*). We divided our analysis window into early and late-phase spiking activity, postulating that the late inhibition created by Pyr→SST synaptic properties would differentially contribute to the late versus early network activity profile in L2/3. In response to whisker touch, SST interneurons are recruited more slowly than PV and other interneuron types (Yu et al., 2019). In accordance, in an auditory stimulus adaptation paradigm, PV-mediated adaptation was restricted to the first 50 ms poststimulus (Natan et al., 2017). Conversely, half-maximal recruitment for L2/3 SST interneurons *in vitro* requires at least three stimuli at 50 Hz (Stachniak et al., 2019). We therefore examined the impact of Pyr→SST facilitation over a window for early (0–50 ms) and late phases (50–100 ms) after whisker deflection at 10 Hz, reasoning that removal of Pyr→SST facilitation might selectively alter later portions of evoked spiking patterns *in vivo* (Fig. 6*B*).

To evaluate this, we recorded spiking activity from barrel cortex in response to repetitive single-whisker stimulation at 10 Hz. Previous *in vitro* work shows that high-frequency stimulation (25+ Hz) preferentially recruits SST interneuron type inhibition (Pouille and Scanziani, 2004). Consistent with this, we find that although Pyr→SST synapses showed robust responses to NS102 at 50 Hz stimulation *in vitro*, they did not show responses at lower frequencies (10 Hz), regardless of testing order (P21 normalized change in EPSC amplitude, 50 Hz, NS102, −16.3 ± 5.3% for EPSC3, −20.2 ± 3.3% for EPSC4, and −19.3 ± 2.0% for EPSC5, *p* = 0.02, *p* = 0.001, and *p* = 0.003, respectively; vs baseline, 10 Hz, NS102, −4.0 ± 4.4 for EPSC4, −6.7 ± 3.7 for EPSC5, *p* = 1 and *p* = 1, respectively; vs baseline, *n*/*N* = 7/2, two-way ANOVA with Bonferroni *post hoc*; Fig. 6*C*,*D*). NS102 selectively impaired the late facilitation ratio for 50 Hz stimulation but not at 10 Hz stimulation (P21, EPSC2/EPSC1 ratio, 50 Hz, *p* = 0.4, *t* statistic = 0.90, df = 6, paired *t* test; Fig. 6*E*; EPSC5/EPSC1 ratio, 50 Hz, *p* = 0.009, *t* statistic = 3.81, df = 6, paired *t* test, Fig. 6*F*; EPSC2/EPSC1 ratio, 10 Hz, *p* = 0.5, *t* statistic = 0.70, df = 6, paired *t* test; Fig. 6*G*; EPSC5/EPSC1 ratio, 10 Hz, *p* = 1, *t* statistic = 0.04, df = 6, paired *t* test; Fig. 6*H*). These results suggest that deflecting the whiskers at 10 Hz should not be expected to produce direct recruitment of SST cells, which might have influenced the early window (0–50 ms) after deflection.

It has previously been shown that hippocampal SST-specific knockdown or global removal of *Elfn1* reduces facilitation of excitatory inputs by abolishing presynaptic mGluR7 and GluK2-KAR activity (Sylwestrak and Ghosh, 2012; Stachniak et al., 2019). We used *Elfn1* knock-out animals (*Elfn1 KO*) to remove the facilitation caused by these components *in vivo* (Fig. 6*I*). We first verified that L2/3 Pyr→SST synapses in P21 *Elfn1 KO* animals show reduced facilitation relative to age-matched controls *in vitro* (Fig. 6*J*). Both early and late facilitation are reduced, consistent with the loss of both mGluR7 and GluK2-KARs (EPSC2/EPSC1 at P21, 1.46 ± 0.09 vs 2.31 ± 0.18 in WT, *p* = 0.0002, *t* statistic = 4.56, df = 21, paired *t* test; Fig. 6*K*; EPSC5/EPSC1 at P21, 1.11 ± 0.14 vs 4.74 ± 0.94 in WT, *n*/*N* = 13/4, *n*/*N* = 10/3, corrected *p* = 0.0006, *t* statistic = 4.35, df = 21, paired *t* test; Fig. 6*L*).

Stimulus adaptation was quantified by normalizing average spike rates (baseline subtracted) in the early and late time windows to initial stimulus responses (Fig. 6*M*). We first assessed baseline neural activity and initial stimulus responses in *Elfn1* WT and KO animals, as *Elfn1 KO* mice have some tendency toward epilepsy after 3 months of age, although with normal neuroanatomical development (Dolan and Mitchell, 2013; Tomioka et al., 2014). Baseline neural activity did not differ between *Elfn1 KO* and WT littermates in L2/3 of barrel cortex at P21 (action potentials in 50 ms, L2/3 baseline, 0.017 ± 0.004 in *Elfn1 KO* vs 0.011 ± 0.002 in WT, *p* = 0.3, *t* statistic = 1.18, df = 23, *t* test; Fig. 6*N*). Likewise, the sensory responses to initial whisker deflection did not differ between WT and KO (L2/3 initial response, early, 0.11 ± 0.02 in *Elfn1 KO* vs 0.13 ± 0.01 in WT, *p* = 0.4, *t* statistic = 0.91, df = 23, *t* test; L2/3 initial response, late, 0.09 ± 0.02 in *Elfn1 KO* vs 0.07 ± 0.01 in WT, *p* = 0.5, *t* statistic = 0.67, df = 23, *t* test; Fig. 6*O*). Baseline neural activity also did not differ in L4 at P21 (L4 baseline, 0.029 ± 0.004 in *Elfn1 KO* vs 0.023 ± 0.004 in WT, *p* = 0.3, *t* statistic = 0.96, df = 23, *t* test; Fig. 6*P*) nor did initial whisker responses (L4 initial response, early, 0.13 ± 0.01 in *Elfn1 KO* vs 0.16 ± 0.009 in WT, *p* = 0.06, *t* statistic = 1.97, df = 23, *t* test; L4 initial response, late, 0.13 ± 0.01 in *Elfn1 KO* vs 0.10 ± 0.01 in WT, *p* = 0.2, *t* statistic = 1.46, df = 23, *t* test; Fig. 6*Q*). The loss of mGluR7 from Pyr→SST synapses predicted to accelerate recruitment of SST inhibition (Stachniak et al., 2019), and thus perhaps to reduce initial response amplitude. Although we did not detect significant decreases in early spiking that we could attribute to the loss of mGluR7 effects, strong feedforward activation during whisker stimulation may mask any such effects in the early response window.

In accordance with the developmental transition of presynaptic GluK2 from L4 Pyr→L4 SST to L2/3 Pyr→L2/3 SST synapses observed *in vitro*, we expected that at P21, *Elfn1* removal would have a more pronounced effect in L2/3. We find that repetitive whisker stimulation evoked sensory adaptation in both WT and KO animals (late adaptation vs initial response in L2/3, WT, *p* = 9 × 10^−35^; *Elfn1 KO*, *p* = 3 × 10^−24^; early adaptation vs initial response in L2/3, WT, *p* = 4 × 10^−7^; *Elfn1 KO*, *p* = 1 × 10^−8^, Bonferroni *post hoc*). However, in *Elfn1 KO* mice, L2/3 barrel cortex shows selective impairment of late but not early adaptation to repetitive whisker stimulation compared with control littermates at P21 (late adaptation, 78 ± 5% in *Elfn1 KO* vs 99 ± 6% in WT, *p* = 0.01; early adaptation, 34 ± 7% in KO vs 39 ± 5% in WT, *p* = 1, *n*/*N* = 14/4 WT, *n*/*N* = 11/4 KO, two-way ANOVA, Bonferroni *post hoc*; Fig. 6*R*). In contrast, our characterization of L4 Pyr→L4 SST synapses at P21 showed an absence of detectable GluK2, which led us to speculate that *Elfn1 KO* might not have an impact on late adaptation in L4 as it does in L2/3. Indeed, L4 barrel cortex did not show a significant change in adaptation in response to *Elfn1* removal (late adaptation, 85 ± 8% in KO vs 94 ± 6% in WT, *p* = 1; early adaptation, −1 ± 7% in KO vs 10 ± 5% in WT, *p* = 1, *n*/*N* = 14/4 WT, *n*/*N* = 11/4 KO, two-way ANOVA, Bonferroni *post hoc*; Fig. 6*S*). Interestingly, unlike L2/3, L4 did not show substantial early-phase adaptation, but still expressed prominent late-phase adaptation for both WT and KO (late adaptation vs initial response, WT, *p* = 3 × 10^−28^; *Elfn1 KO*, *p* = 6 × 10^−19^; early adaptation vs initial response, WT, *p* = 1; *Elfn1 KO*, *p* = 0.7; Bonferroni *post hoc*). The absence of adaptation in the early phase may be attributed to strong feedforward activation of L4 barrel cortex by thalamocortical afferents, which could indicate that the feedforward transfer of sensory information in response to whisker touch is intact in the KO mice.

**Figure 7.**
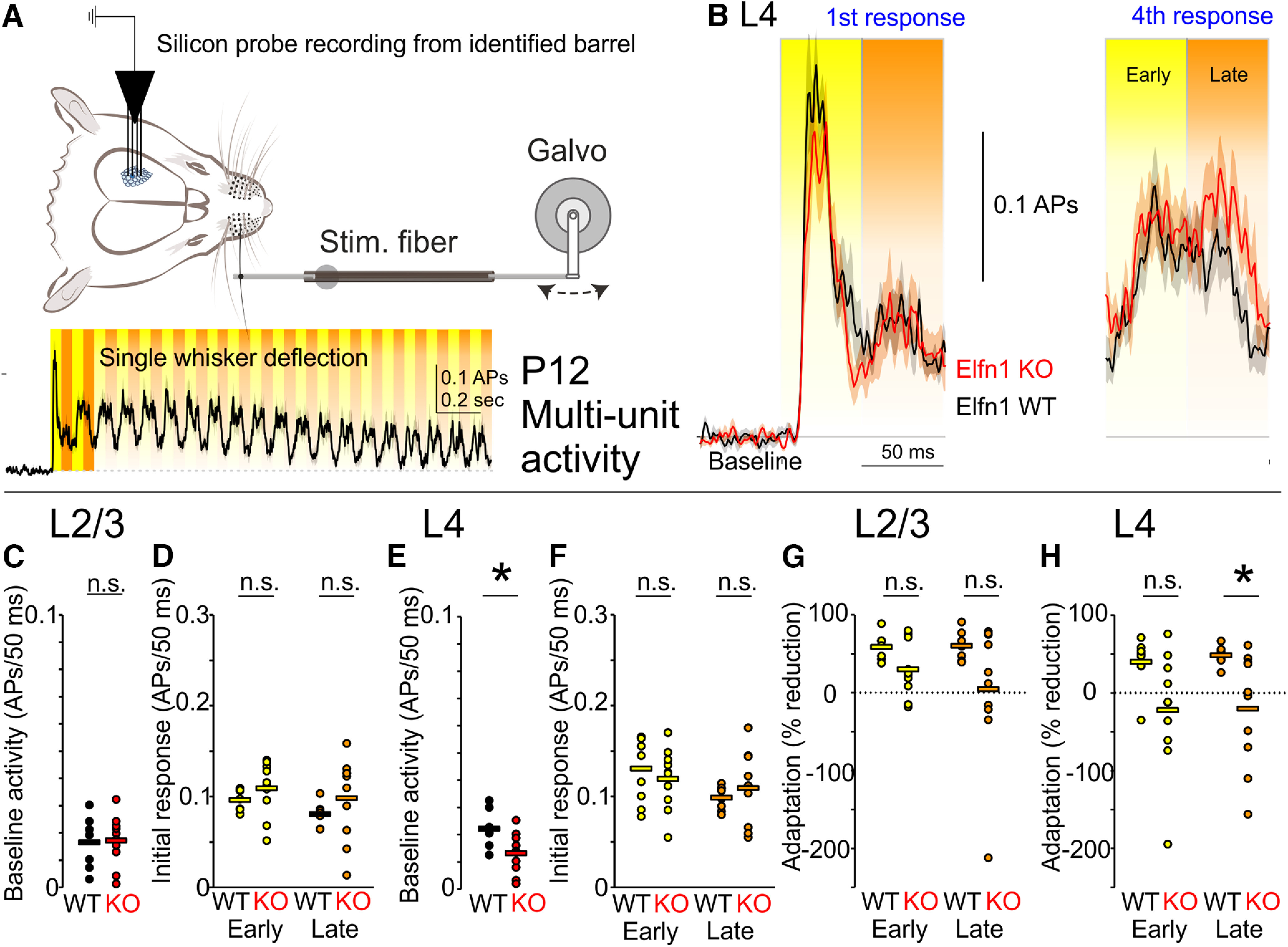
L4 Late adaptation is impaired in *Elfn1 KO* at P12. ***A***, Silicon probe multiunit activity from L4 of identified barrels at P12 in response to repetitive single-whisker deflection. ***B***, Left, Initial responses to whisker deflection are similar in *Elfn1 WT* and *KO*. Right, Late attenuation is reduced with subsequent deflections (fourth pulse) in *Elfn1 KO*. Traces are mean ± SEM. ***C***, Baseline activity in L2/3 barrel cortex does not differ between WT and KO at P12. ***D***, Initial responses in L2/3 to whisker touch do not differ between WT and KO. ***E***, Baseline activity in L4 is reduced in *Elfn1 KO*. ***F***, Initial responses in L4 do not differ. ***G***, At P12, neither early nor late adaptation are significantly reduced in L2/3 KO. ***H***, In L4, late-phase activity shows a significant reduction in adaptation in *Elfn1 KO*; n.s., *p* > 0.05, *p < 0.05.

Based on our *in vitro* findings, we further speculated that the observed changes in late adaptation would be apparent in L4 of cortex at an earlier point in development. We selected P11–P13, as whisker-evoked cortical responses are reliable by this time (Fig. 7*A*,*B*), but active whisking has not yet emerged (Cai et al., 2022). At P12, baseline neural activity and initial whisker responses did not differ between *Elfn1 KO* and WT littermates in L2/3 of barrel cortex (action potentials in 50 ms, L2/3 baseline, 0.017 ± 0.004 in *Elfn1 KO* vs 0.017 ± 0.003 in WT, *p* = 0.9, *t* statistic = 0.15, df = 15, *t* test; Fig. 7*C*; L2/3 initial response, early, 0.11 ± 0.009 in *Elfn1 KO* vs 0.10 ± 0.004 in WT, *p* = 0.2, *t* statistic = 1.09, df = 15, *t* test; L2/3 initial response, late, 0.10 ± 0.01 in *Elfn1 KO* vs 0.08 ± 0.005 in WT, *p* = 0.3, *t* statistic = 0.99, df = 15, *t* test; Fig. 7*D*). In L4, baseline activity was reduced in *Elfn1 KO*, whereas evoked responses did not differ (action potentials in 50 ms, L4 baseline, 0.013 ± 0.002 in *Elfn1 KO* vs 0.022 ± 0.003 in WT, *p* = 0.02, *t* statistic = 2.50, df = 15, *t* test; Fig. 7*E*; L4 initial response, early, 0.12 ± 0.01 in *Elfn1 KO* vs 0.14 ± 0.01 in WT, *p* = 0.3, *t* statistic = 1.05, df = 15, *t* test; L4 initial response, late, 0.11 ± 0.01 in *Elfn1 KO* vs 0.11 ± 0.01 in WT, *p* = 1, *t* statistic = 0.03, df = 15, *t* test; Fig. 7*F*). In contrast to P21, *Elfn1 KO* mice only trended toward a reduction in late adaptation in L2/3 at P12, not reaching significance, albeit sensory adaptation was not as significantly expressed at this age (early adaptation, 30 ± 11% in KO vs 59 ± 7% in WT, *p* = 1; late adaptation, 5 ± 28% in *Elfn1 KO* vs 60 ± 7% in WT, *p* = 0.1, *n*/*N* = 7/3 WT, *n*/*N* = 10/4 KO, two-way ANOVA, Bonferroni *post hoc*; Fig. 7*G*; early adaptation vs initial response, WT, *p* = 0.1; *Elfn1 KO*, *p* = 1; late adaptation vs initial response, WT, *p* = 0.1; *Elfn1 KO*, *p* = 1; Bonferroni *post hoc*). Late adaptation was nevertheless reduced in L4 in P12 *Elfn1 KO* mice (early adaptation, −22 ± 24% in KO vs 40 ± 13% in WT, *p* = 0.1; late adaptation, −20 ± 23% in *Elfn1 KO* vs 48 ± 5% in WT, *p* = 0.0495, *n*/*N*= 7/3 WT, *n*/*N*= 10/4 KO, two-way ANOVA, Bonferroni *post hoc*; Fig. 7*H*) As with L2/3, L4 does not appear to express significant levels of sensory adaptation at P12 (early adaptation vs initial response, WT, *p* = 1; *Elfn1 KO*, *p* = 1; late adaptation vs initial response, WT, *p* = 1; *Elfn1 KO*, *p* = 1, Bonferroni *post hoc*). One should note that a multitude of changes are taking place in the cortex at this early time point as illustrated by the changes in sensory-driven cortical activity compared with a week later, which includes a less reliable adaptation in both layers examined. Nonetheless, our results support a transfer of late-phase adaptation from L4 to L2/3 as cortex matures from P12 to P21.

**Figure 8.**
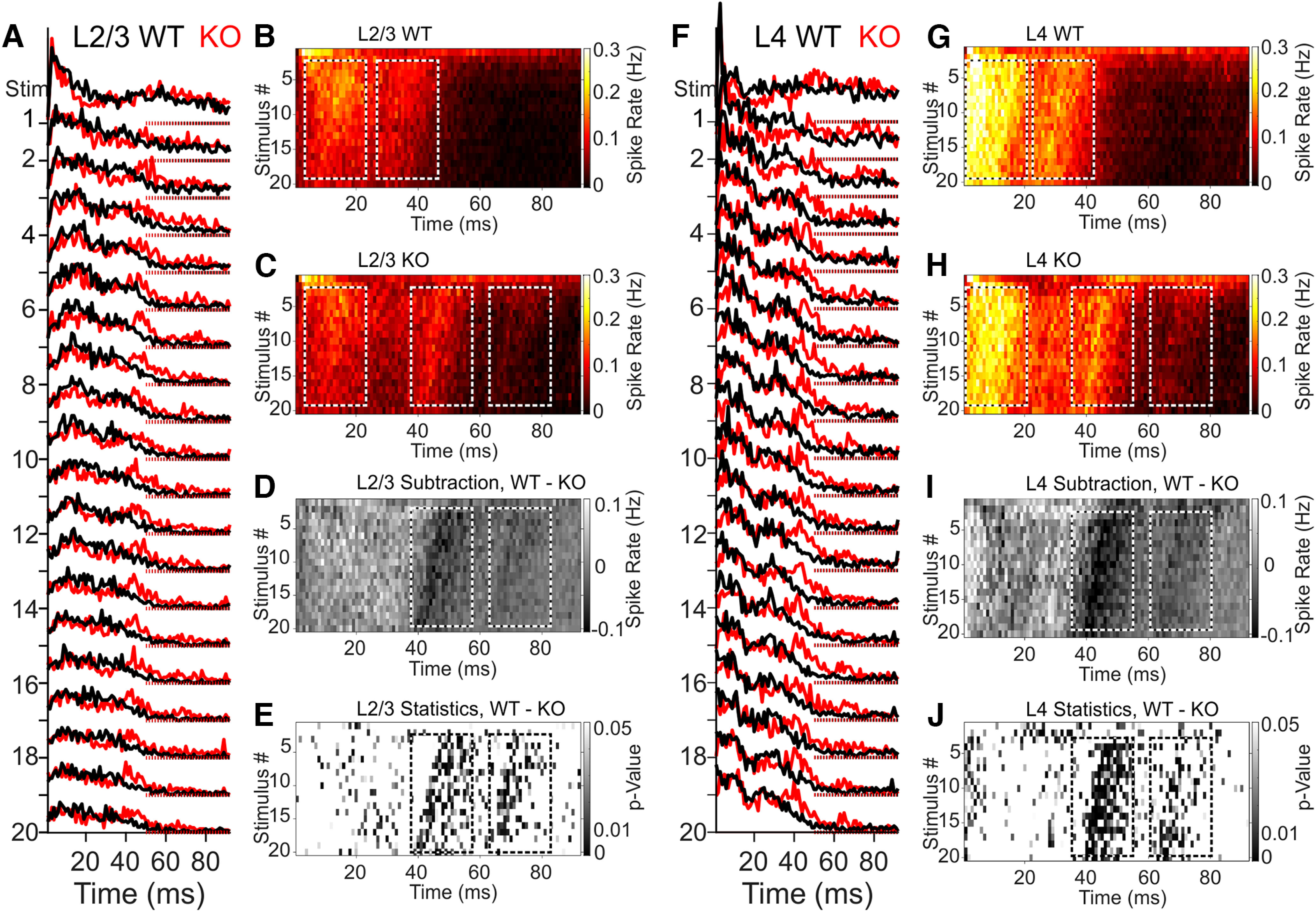
*Elfn1 KO* shifts the timing of whisker-evoked activity and releases high-frequency spiking. ***A***, Smoothed multiunit activity in response to stimulation pulse 1–20 (20 pulses in 2 s) averaged across barrels for L2/3 WT (black, n/*N* = 13/4) and KO (red, *n*/*N* = 11/4). Dashed lines indicate baseline. ***B***, MUA heat maps of 20 pulses averaged across WT L2/3 recordings, as in ***A***, show nearly complete adaptation in the late phase. ***C***, KO animals show substantial late-phase activity, in waves. ***D***, Subtracting WT L2/3 MUA and KO L2/3 MUA average heat maps, we find marginal change in the early window but substantial alterations in late-phase activity. ***E***, Spatiotemporal distribution of *p* values for WT versus *Elfn1 KO*, with thresholding at *p* = 0.05. Darker colors represent higher significance. Waves of differential activity are apparent at 20 ms intervals (i.e., 50 Hz). ***F–J***, Same as for ***A–E*** for L4.

To further determine how differences in adaptation develop over time, we examined the average spiking responses across the stimulus train at P21, where the response to each deflection was well constrained within the 100 ms window (20 deflections in 2 s; Fig. 8*A*,*F*). We then created instantaneous firing rate heat plots, averaged from WT (Fig. 8*B*,*G*) and KO (Fig. 8*C*,*H*) mice for the 100 ms interval following each deflection. Two prominent bands of activity were visible in the distributions of WT and KO heat plots (Fig. 8, boxed regions), which advanced to earlier time points with an increasing stimulus number. Although we found little change for the first band in either L2/3 or L4, *Elfn1 KO* mice showed a shift of the second band of activity to ∼10 ms later after deflection compared with WT. This difference is apparent in the subtracted activity maps (Fig. 8*D*,*I*) and the associated point-by-point changes in the *p* value for both layers (Fig. 8*E*,*J*). This comprehensive representation of the data further revealed that the late-phase activity of both layers displays an additional distinct band of recurring spiking, separated by ∼20 ms (50 Hz; Fig. 8, boxed regions). Synaptic facilitation at Pyr→SST synapses thus allows for feedback suppression of high-frequency cortical activity. Although these *in vivo* findings are consistent with our *in vitro* results, it is important to consider that cortical layers do not behave independently, and thus the late-phase activity we measure will include influences from multiple cortical layers.

## Discussion

### Dynamic recruitment of presynaptic KARs at cortical Pyr→SST synapses across development

Cortical GABAergic interneuron diversity allows for effective inhibitory control over a variety of computational regimes. Delayed inhibition via SST interneurons enables efficacious silencing of a distinct computational compartment located in the distal tufts of cortical pyramidal neurons (Takahashi et al., 2016; Schulz et al., 2018). Presynaptic GluK2-KARs open slowly in response to glutamatergic activation because of a voltage-dependent blockade by intracellular polyamines (Bowie and Mayer, 1995). As a result, GluK2-KARs activity does not affect the early facilitation of Pyr→SST synapses. Bidirectional regulation of GluK2-KARs therefore provides dynamic regulation of the magnitude of inhibitory control granted by SST neurons, without completely sacrificing the frequency-dependent facilitation necessary to produce SST delayed inhibition. Here, we find that recruitment of GluK2 receptors to Pyr→SST synapses is activity dependent. In addition, ongoing maintenance of GluK2-KARs also appears to be activity- and CaMKII-dependent, as whisker trimming or acute application of KN-62 reduces GluK2-KAR activity. In L2/3 barrel cortex, we find an age-dependent recruitment of presynaptic KARs that matures by the end of the second postnatal week. Interestingly, several days of whisker deprivation did not appear to substantially reduce late facilitation in barrel cortex. It should be noted here that whisker deprivation is a relatively crude method of manipulating cortical activity and is likely to evoke multiple changes in synaptic properties, including reductions in initial release probability or homeostatic plasticity (Bender et al., 2006; Glazewski et al., 2017). Alternatively, we note that maturation (from P6 to P14) of excitatory synapses onto hippocampal interneurons includes the loss of tonically active GluK1-containing presynaptic kainate receptors, resulting in a loss in early and late facilitation (Lauri et al., 2006, 2021). Potentially, synaptic maturation may include the exchange of presynaptic GluK1-KARs for GluK2-KARs. Indeed, the activity-dependent downregulation of presynaptic GluK1-KARs at immature CA3→CA1 glutamatergic synapses involves a change in KAR affinity, suggestive of a change in receptor subunit composition (Lauri et al., 2006). However, BDNF-dependent developmental downregulation of GluK1-KARs is irreversible and closes a developmental window for presynaptic plasticity (Clarke et al., 2014). In contrast, we find that the maturation of GluK2-KAR activity is not a hard-wired genetic program restricted to a particular developmental window, as whisker trimming late in development still impedes GluK2-KAR activity. This suggests that regulation of Pyr→SST synaptic strength, and therefore recruitment of SST inhibition, could be tuned on demand. As manipulations that upregulate GluK2 appear to downregulate GluK1 and vice versa, it may be conjectured that during dynamic scaling of Pyr→SST synaptic strength, the facilitation ratio can be defended through the selective regulation of subunit composition of presynaptic kainate receptors. An alternative hypothesis would be a change in the phosphorylation state of the kainate receptors that alters receptor activity or NS102 responsiveness without affecting subunit composition. Given the relatively long time course for modulation *in vitro* (15–25 min) we cannot distinguish between these mechanisms at present. In contrast, L5 Pyr→SST synapses do not appear to stably express GluK2-KARs (Stachniak et al., 2019). If dynamic recruitment dictates the presence or absence of receptors, perhaps this divergent observation is best explained by the fact that these experiments were performed in young juvenile mice (P11–P19), and the absence in L5 SST cells could correspond to a reserve pool of recruitable inhibition that could be engaged as the mouse matures. It is therefore interesting to note that the developmental increase in L2/3 presynaptic GluK2-KARs coincides with a dramatic increase in cortical excitability that precedes the onset of active whisking (Cai et al., 2022). During the third postnatal week, active whisking is established, and KARs are reduced at L4 Pyr→SST synapses. This time window therefore corresponds to an age where active internalization of sensory representations is ongoing, and a switch from passive sensory experience to active interrogation of the tactile environment is occurring. Our observations support the conjecture from previous studies suggesting that internal representations are communicated to the distal tufts of pyramidal neurons and that synchrony between internal (tuft) and external (perisomatic) representations are critical for the learning process (Doron et al., 2020).

### Pyr→SST synaptic facilitation contributes to late-phase sensory adaptation

Our evaluation of whisker-evoked responses in WT and *Elfn1 KO* animals suggests that the short-term dynamics of inputs to somatostatin neurons and the subsequent delayed recruitment of these cells contribute to the late-phase suppression of repetitive sensory information. Further, we find that this phenomenon tracks with the maturation of sensory processing across layers. Bearing in mind that evoked sensory responses change dramatically over the first several postnatal weeks (van der Bourg et al., 2019; Cai et al., 2022), we do find that Elfn1 recruitment of presynaptic KARs can affect late adaptation, first in L4 at P12, followed by L2/3 at P21. Allowing for the prominent differences observed in early adaptation, this is nonetheless in good concordance with the sequential maturation of SST cells in these layers, as indicated by presynaptic KARs *in vitro*.

Perception of whisker touch requires the influx of sensory information into the distal tufts of subcortically projecting L5 pyramidal neurons and their late phase of spiking activity (Sachidhanandam et al., 2013; Takahashi et al., 2020). Extraneous sensory information could therefore be ignored by inhibiting L1 dendrites of pyramidal neurons. The late inhibition recruited through SST interneurons is therefore both spatially and temporally suited to produce suppression of extraneous sensory stimuli, such as occurs with repetitive whisker touches. Indeed, SST-mediated inhibition of dendritic tufts can reduce perception of whisker touch *in vivo* (Takahashi et al., 2016) Further, SST neuron activation in response to visual stimuli and also SST-mediated sensory adaptation to auditory stimuli are specific to the presentation of repeated stimuli (Natan et al., 2017; Hayden et al., 2021). Here, we find that eliminating SST facilitation with *Elfn1* deletion results in an increase in the late-phase cortical activity of putative pyramidal cells during sensory adaptation, consistent with a reduction in late inhibition. By comparison, acute inhibition of SST cells during auditory stimulation likewise increased late-phase activity but only for adapted and not for unadapted stimuli (Natan et al., 2017). In contrast, optogenetic activation of either SST or PV neurons produced only nonselective inhibition of auditory responses (Natan et al., 2017). The divergence in the outcomes of these experimental paradigms indicates that cell-specific synaptic properties, including short-term facilitation, play a critical role in shaping the inhibitory regimes governed by these diverse interneuron classes.

Intriguingly, the emergence of high-frequency phasic activity in the *Elfn1 KO* suggests that Pyr→SST facilitation tunes SST interneurons to regulate a discrete type of cortical activity that includes high-frequency firing. Thus, it appears that the activity-dependent recruitment and maintenance of presynaptic GluK2-KARs provides a means to dynamically regulate late-phase high-frequency responses, scaled to cortical activity. Of note, Elfn1 also produces facilitation onto VIP multipolar interneurons at the L1/L2 border, albeit to a much lesser extent than to SST neurons, and so *Elfn1* deletion from VIP cells may also contribute to the loss of late-phase inhibition in L2/3 (Stachniak et al., 2021). Although a role for GluK2-KARs in VIP multipolar facilitation has not been established, multipolar VIP hippocampal cells have been shown to preferentially synapse onto pyramidal neurons rather than interneurons (Tyan et al., 2014).

### Potential relevance of SST cell short-term dynamics to sensory deficits and disease

Interestingly, failure to suppress neural responses to repeated auditory stimuli is a hallmark characteristic of neurodevelopmental conditions such as schizophrenia, fragile X, and autism (Orekhova et al., 2008; Ethridge et al., 2016; San-Martin et al., 2020). Intriguingly, recent analysis of single-cell RNAseq data from autism and control populations has localized substantial autism risk gene expression profiles to SST interneurons (Velmeshev et al., 2019). Furthermore, the risk genes that most closely correlated to clinical severity were also enriched in SST interneurons (Velmeshev et al., 2019). In light of the predictive value of sensory gating clinical diagnostics (Minassian et al., 2007), any changes in SST neuron activity or the ability to dynamically regulate late inhibition with GluK2-KAR may influence the severity of clinical symptoms. Here, we find that late inhibition contributes to suppression of cortical responses to repeated stimuli, suggesting that sensory gating deficits may represent an inability to dynamically regulate late inhibition and mitigate cortical hyperexcitability. This proposition is supported by observations that *Elfn1*, *mGluR7*, and *GluK2* are risk factors for schizophrenia/autism (Micheau et al., 2014; Guzmán et al., 2017; Thyme et al., 2019; Fisher et al., 2020, 2021). In summary, we find that experience-dependent maturation of cortical inhibition includes the adaptive enhancement of Pyr→SST synapses with GluK2 KARs.
